# Yiguanjian alleviates ocular surface inflammation and lacrimal gland immune dysregulation in experimental dry eye disease: evidence from *in vivo* and *in vitro* models

**DOI:** 10.3389/fmed.2026.1915720

**Published:** 2026-09-28

**Authors:** Yuan Zhong, Jian Shi, Xiyuan Liu, Yanwen Zhang, Qinghua Peng, Jun Peng

**Affiliations:** 1 The First Hospital of Hunan University of Chinese Medicine, Changsha, China; 2 Hunan University of Chinese Medicine, Changsha, China

**Keywords:** dry eye disease, epithelial–immune crosstalk, lacrimal gland, ocular surface inflammation, Th17/Treg balance, Yiguanjian

## Abstract

**Background:**

Persistent ocular surface inflammation and lacrimal gland immune dysregulation are central to the progression of dry eye disease (DED). Th17/Treg imbalance contributes to sustained inflammatory activation in the ocular surface and lacrimal gland. Yiguanjian (YGJ) has anti-inflammatory and immunomodulatory potential, but whether it can alleviate ocular surface inflammation and restore lacrimal gland immune homeostasis in DED remains unclear.

**Methods:**

A benzalkonium chloride (BAC)-induced rat DED model was used to assess the effects of YGJ on tear secretion, tear film break-up time, corneal fluorescein staining, ocular histopathology, lacrimal gland Th17/Treg proportions, related gene expression, and STAT3 signaling. Yiguanjian-containing serum (YCS) was prepared, and its serum-associated chemical features were characterized by UHPLC-Q-Orbitrap HRMS. A hyperosmotic non-contact co-culture system of rat lacrimal gland epithelial cells and activated CD4⁺ T cells was established to evaluate epithelial cell viability, intracellular reactive oxygen species (ROS), Th17/Treg differentiation, Foxp3 and IL-17A immunofluorescence signals, and related gene expression. AS2863619 and Peptide P60 were used to explore Foxp3-associated regulation.

**Results:**

In BAC-induced dry eye rats, YGJ improved tear secretion and tear film stability, reduced corneal fluorescein staining, and alleviated corneal and lacrimal gland histopathological injury, with partial preservation of meibomian gland and conjunctival morphology. YGJ reduced lacrimal gland Th17 proportions, restored Treg proportions, increased Foxp3 and IL-10 expression, decreased RORγt and IL-17A expression, and reduced lacrimal gland STAT3 phosphorylation. UHPLC-Q-Orbitrap HRMS yielded 67 candidate annotations corresponding to 56 distinct LC–MS features in YCS. In the hyperosmotic co-culture model, YCS improved epithelial cell viability, reduced ROS accumulation in CD4⁺ T cells, suppressed Th17-related responses, and enhanced Treg-associated immunoregulation. AS2863619 produced effects comparable to YCS, whereas Peptide P60 weakened Foxp3-associated immunoregulation; combined YCS treatment partially attenuated Peptide P60-induced changes.

**Conclusion:**

YGJ attenuated DED-related inflammatory injury *in vivo* and modulated lacrimal gland epithelial cell–CD4⁺ T-cell inflammatory interactions *in vitro*. These findings were accompanied by reduced lacrimal gland STAT3 phosphorylation *in vivo* and lower ROS accumulation in CD4⁺ T cells *in vitro*, together with Foxp3-associated improvement of the Th17/Treg balance; however, the causal relationship between the STAT3 and ROS findings was not established.

## 1 Introduction

Dry eye disease (DED) is a multifactorial ocular surface disorder characterized by tear film instability, tear hyperosmolarity, ocular surface inflammation, epithelial injury, and neurosensory abnormalities (1). Although DED may arise from diverse etiological factors, persistent inflammation and immune dysregulation are central events that drive disease progression and chronicity (2). Tear hyperosmolarity can injure ocular surface epithelial cells and may contribute to inflammatory activation within the lacrimal functional unit, including the lacrimal gland, thereby promoting the release of inflammatory mediators and amplification of local immune responses. This epithelial–immune interaction contributes to a self-sustaining inflammatory microenvironment and further aggravates ocular surface damage.

Among the immune mechanisms implicated in DED, imbalance between T helper 17 (Th17) cells and regulatory T cells (Treg) has emerged as an important feature of chronic ocular surface inflammation (2, 3). Excessive Th17 responses promote the production of pro-inflammatory cytokines, particularly interleukin-17A (IL-17A), thereby aggravating epithelial barrier disruption and tissue inflammation. In contrast, Treg cells are essential for maintaining immune tolerance and limiting excessive inflammatory activation. Impaired Treg differentiation or function may weaken immune restraint and favor persistent inflammation. Therefore, restoring the Th17/Treg balance represents a relevant strategy for controlling DED-related immune inflammation.

The Th17/Treg axis is regulated by multiple transcriptional and signaling mechanisms. Forkhead box P3 (Foxp3) is a key transcription factor required for Treg differentiation and immunosuppressive function, whereas retinoic acid receptor-related orphan receptor gamma t (RORγt) is closely associated with Th17 differentiation and IL-17A-mediated inflammatory responses. Signal transducer and activator of transcription 3 (STAT3) is also closely involved in Th17-related inflammatory polarization and may contribute to the disruption of Th17/Treg homeostasis. In addition, oxidative stress is closely linked to epithelial injury and immune-cell polarization in DED (4, 5). Excessive reactive oxygen species (ROS) accumulation can intensify inflammatory signaling and further disturb immune homeostasis. These pathways may interact under hyperosmotic stress and provide a mechanistic basis for investigating immune regulation and epithelial–immune inflammatory crosstalk in DED.

Yiguanjian (YGJ) is a classical traditional Chinese medicine formula traditionally used to nourish yin, enrich blood, soothe the liver, and regulate qi (6). Modern pharmacological studies have suggested that YGJ and its related bioactive constituents possess anti-inflammatory, antioxidant, and immunomodulatory properties (7). These pharmacological features support the rationale for evaluating YGJ in inflammatory ocular surface disorders involving epithelial injury and immune imbalance. However, whether YGJ can alleviate DED-related ocular surface injury and restore lacrimal gland immune homeostasis remains insufficiently defined. Moreover, because orally administered herbal formulae undergo absorption and metabolic transformation and may also induce endogenous metabolic changes *in vivo*, characterizing the serum-associated chemical profile after YGJ administration is important for investigating the circulating factors potentially involved in epithelial–immune inflammatory regulation.

In the present study, a benzalkonium chloride (BAC)-induced rat model of DED was established to evaluate the effects of YGJ on tear secretion, tear film stability, corneal epithelial injury, ocular adnexal histopathology, lacrimal gland Th17/Treg imbalance, and STAT3 signaling *in vivo*. Yiguanjian-containing serum (YCS) was then prepared, and its serum-associated chemical profile was characterized using ultra-high-performance liquid chromatography–Q-Orbitrap high-resolution mass spectrometry (UHPLC-Q-Orbitrap HRMS). To further explore epithelial–immune interactions under DED-related stress, a non-contact co-culture model of rat lacrimal gland epithelial cells and activated CD4⁺ T cells was established under hyperosmotic conditions. By integrating an experimental DED model, serum pharmacochemistry, and a hyperosmotic lacrimal gland epithelial cell–CD4⁺ T-cell co-culture system, this study aimed to determine whether YGJ alleviates ocular surface inflammation and lacrimal gland immune dysregulation by restoring Th17/Treg homeostasis and modulating epithelial–immune inflammatory crosstalk.

## 2 Materials and methods

### 2.1 In *vivo* experiment

#### 2.1.1 Animals, grouping, and BAC-induced dry eye model

Sixty healthy male Sprague–Dawley (SD) rats aged 8 weeks were obtained from Hunan SJA Laboratory Animal Co., Ltd. [Changsha, China; animal production license No. SCXK (Xiang) 2024-0014; certificate No. ZS-202505060008]. All animals were housed in the barrier facility of the Experimental Animal Center of Hunan University of Chinese Medicine under controlled conditions, with a temperature of 23 ± 2° C and relative humidity of 56 ± 4%, and had free access to food and water. The animal facility was approved under license No. SYXK [Xiang] 2021-0002. All experimental procedures were reviewed and approved by the Animal Ethics Committee of Hunan University of Chinese Medicine (Approval No. HNUCM21-2505-04).

After one week of acclimatization, the rats were randomly divided into a Control group (*n* = 10) and a modeling group (*n* = 50). Rats in the modeling group received topical instillation of 0.2% benzalkonium chloride (BAC; 5 μL per eye), twice daily at 9:00 and 17:00, for 14 consecutive days to induce experimental dry eye disease (DED) (8). Model establishment was evaluated on day 14 based on ocular surface parameters commonly used in BAC-induced dry eye models, including tear secretion measurement and corneal fluorescein staining. Successful modeling was defined as reduced tear secretion, shortened tear film break-up time, and increased corneal fluorescein staining compared with baseline values and the Control group, indicating the establishment of an experimental dry eye phenotype. Successfully modeled rats were then randomly assigned to five groups: Model group, sodium hyaluronate group, low-dose YGJ group (YGJ-L, 2.93 g/kg), medium-dose YGJ group (YGJ-M, 5.85 g/kg), and high-dose YGJ group (YGJ-H, 11.7 g/kg), with 10 rats in each group. The corresponding interventions were administered for 28 consecutive days.

Rats in the Control and Model groups received an equal volume of distilled water by gavage once daily. Rats in the sodium hyaluronate (SH) group received 0.3% sodium hyaluronate eye drops (Zhenshiming Pharmaceutical Co., Ltd., China; Batch No. 250606), one drop per eye once daily for 28 consecutive days. Rats in the YGJ groups received the corresponding dose of YGJ decoction by gavage once daily for 28 consecutive days. After the final administration and ocular surface assessments, rats were deeply anesthetized with pentobarbital sodium, followed by abdominal aortic blood collection. Death was confirmed before tissue collection. Corneal, conjunctival, meibomian gland, and lacrimal gland tissues were then collected for subsequent analyses. Corneal, conjunctival, meibomian gland, and lacrimal gland tissues were allocated for histological staining, flow cytometry, western blotting, or RT-qPCR according to the requirements of each assay. Samples used for histological staining were fixed, samples used for flow cytometry were processed immediately as fresh tissues, and samples used for molecular assays were stored at −80° C.

#### 2.1.2 Tear secretion test, tear film break-up time, and corneal fluorescein staining

Tear secretion and tear film break-up time were assessed at baseline, after BAC modeling, and after completion of treatment. Corneal fluorescein staining was performed after BAC modeling and after treatment to evaluate corneal epithelial injury. Rats were anesthetized with 2% isoflurane before ocular surface assessments.

Tear secretion was measured using phenol red thread. Briefly, the phenol red thread was placed at the junction of the middle and lateral third of the lower eyelid conjunctiva. After 20 s, the thread was removed, and the length of the red-stained portion was measured.

Tear film break-up time was measured after sodium fluorescein instillation. After fluorescein was evenly distributed over the ocular surface by gently inducing blinking three times, the time from the last blink to the first appearance of a dry spot on the corneal surface under cobalt blue light was recorded.

For corneal fluorescein staining, sodium fluorescein eye drops were instilled into the conjunctival sac. To ensure even distribution of fluorescein over the corneal surface, blinking was gently induced three times using microsurgical forceps. After 90 s, corneal staining was examined under cobalt blue illumination using a slit-lamp microscope at ×40 magnification. All examinations were performed by the same investigator under consistent environmental conditions.

#### 2.1.3 Histological staining

Corneal, lacrimal gland, conjunctival, and meibomian gland tissues were fixed, dehydrated through graded ethanol, cleared in xylene, embedded in paraffin, and sectioned at a thickness of 3–5 μm. Hematoxylin and eosin (HE) staining was performed to evaluate histopathological changes in corneal, lacrimal gland, and meibomian gland tissues. After routine deparaffinization and rehydration, sections were stained with hematoxylin, differentiated with 1% hydrochloric acid alcohol, counterstained with eosin, dehydrated, cleared, and mounted. Histological changes were observed under a light microscope.

Periodic acid–Schiff (PAS) staining was performed to visualize PAS-positive mucin-associated regions along the conjunctival epithelium. After routine deparaffinization and rehydration, conjunctival sections were stained according to the standard PAS staining procedure. The distribution and continuity of PAS-positive staining were evaluated under a light microscope.

#### 2.1.4 Flow cytometric analysis of lacrimal gland Th17 and Treg cells

Fresh lacrimal gland tissues were collected and processed into single-cell suspensions for flow cytometric analysis. Briefly, lacrimal gland tissues were cut into 1–2 mm fragments and digested with a mixed enzyme solution containing 0.125% trypsin and 0.5 mg/mL collagenase II at 37° C for 60 min with shaking at 100 rpm. During digestion, tissue fragments were gently pipetted every 10 min. The cell suspension was filtered through a 70 μm cell strainer and centrifuged at 1200 rpm for 5 min. Red blood cells were lysed using red blood cell lysis buffer, and the remaining cells were collected by centrifugation at 350 × g for 5 min and resuspended for subsequent staining. For Th17 analysis, cells were resuspended in RPMI-1640 medium containing 10% fetal bovine serum (FBS), stimulated with Cell Stimulation Cocktail plus protein transport inhibitors, and incubated at 37° C for 4 h. Cells were then washed with 0.5% bovine serum albumin (BSA)-PBS and stained with antibodies against CD3 (eBioscience™, Cat. No. 11-0030-81) and CD4 (BioLegend, Cat. No. 201507) for 30 min in the dark. After fixation with Intracellular Fixation Buffer and permeabilization with 1 × Permeabilization Buffer, cells were intracellularly stained with anti-IL-17A antibody (eBioscience™, Cat. No. 17-7177-81) for 30 min in the dark.

For Treg analysis, cells were stained with antibodies against CD4 (BioLegend, Cat. No. 201507) and CD25 (eBioscience™, Cat. No. 17-0390-82) for 30 min in the dark. After fixation with Intracellular Fixation Buffer and permeabilization with 1 × Permeabilization Buffer, cells were intracellularly stained with anti-Foxp3 antibody (eBioscience™, Cat. No. 53-5773-82) for 30 min in the dark. Unstained and single-stained controls were included for compensation and gating. After staining, cells were washed with 0.5% BSA-PBS and resuspended for flow cytometric acquisition. Data were acquired using a Beckman flow cytometer (Beckman, Cat. No. A00-1-1102), and the proportions of Th17 and Treg cells in lacrimal gland tissues were analyzed using flow cytometry software. Th17 cells were identified as CD3⁺CD4⁺IL-17A⁺ cells after CD3/CD4 surface staining and intracellular IL-17A staining, whereas Treg cells were identified as CD4⁺CD25⁺Foxp3⁺ cells.

#### 2.1.5 Western blot analysis of STAT3 and Th17/Treg-related proteins in lacrimal gland tissues

Total protein was extracted from lacrimal gland tissues using radioimmunoprecipitation assay (RIPA) lysis buffer supplemented with protease and phosphatase inhibitors. Briefly, tissue samples were washed with pre-cooled phosphate-buffered saline (PBS), cut into small pieces, homogenized in lysis buffer, and lysed on ice. After centrifugation at 12,000 rpm for 10 min at 4° C, the supernatant was collected as the total protein extract. Protein concentration was determined using a bicinchoninic acid (BCA) protein assay kit.

Equal amounts of protein were mixed with loading buffer, denatured, separated by sodium dodecyl sulfate–polyacrylamide gel electrophoresis (SDS-PAGE), and transferred onto polyvinylidene fluoride (PVDF) membranes. After blocking with 5% bovine serum albumin (BSA) at room temperature for 30 min, the membranes were incubated overnight at 4° C with primary antibodies against phosphorylated STAT3 Tyr705 (p-STAT3; CST, Cat. No. 9145; 1:1,000), STAT3 (CST, Cat. No. 9139; 1:1,500), RORγt (Servicebio, Cat. No. GB112204; 1:750), Foxp3 (Proteintech, Cat. No. 85128-6-RR; 1:3,000), and β-actin (Servicebio, Cat. No. GB15003; 1:5,000). After washing with TBST, the membranes were incubated at room temperature with horseradish peroxidase (HRP)-conjugated goat anti-rabbit IgG secondary antibody (Servicebio, Cat. No. GB23303; 1:3,000) for rabbit primary antibodies or HRP-conjugated goat anti-mouse IgG (H+L) secondary antibody (Abiowell, China, Cat. No. AWS0001; 1:5,000) for the mouse anti-STAT3 primary antibody.

Protein bands were visualized using enhanced chemiluminescence (ECL) reagent and captured using a chemiluminescence imaging system. Densitometric analysis was performed using ImageJ software. The expression levels of Foxp3 and RORγt were normalized to β-actin, and phosphorylated STAT3 was normalized to total STAT3.

#### 2.1.6 RT-qPCR analysis of lacrimal gland tissues

Total RNA was extracted from lacrimal gland tissues using RNA extraction reagent (Servicebio, Wuhan, China; Cat. No. G3013) according to the manufacturer's instructions. RNA concentration and purity were measured using a NanoDrop 2000 spectrophotometer. cDNA was synthesized using SweScript All-in-One RT SuperMix for qPCR (Servicebio; Cat. No. G3337). RT-qPCR was performed using 2 × Universal Blue SYBR Green qPCR Master Mix (Servicebio; Cat. No. G3326) on a CFX Connect Real-Time PCR System (Bio-Rad, USA). The amplification conditions were as follows: initial denaturation at 95 °C for 30 s, followed by 40 cycles of denaturation at 95° C for 15 s and annealing/extension at 60° C for 30 s. A melting curve analysis was performed from 65° C to 95° C. β-actin was used as the internal control for lacrimal gland tissue samples, and relative gene expression was calculated using the 2^∧^−ΔΔCt method. The mRNA expression levels of Foxp3, RORγt, IL-17A, and IL-10 in lacrimal gland tissues were detected. Primer sequences for the *in vivo* lacrimal gland tissue experiment are provided in Supplementary Table S1.

### 2.2 Serum pharmacochemistry and *in vitro* epithelial–immune co-culture experiment

#### 2.2.1 Preparation of YGJ decoction and YCS

1. Preparation of YGJ decoction

YGJ decoction consisted of Meliae Fructus (5 g), Ophiopogonis Radix (9 g), Angelicae Sinensis Radix (9 g), Glehniae Radix (12 g), Lycii Fructus (15 g), and Rehmanniae Radix (15 g) (6). All herbs were obtained from the First Hospital of Hunan University of Chinese Medicine (Changsha, China) and met the standards of the Chinese Pharmacopoeia (2020 edition).

Each dose of herbs was decocted twice with 650 mL distilled water for 1 h each time. The combined decoctions were filtered and concentrated to 40 mL, yielding a final concentration of 1.625 g/mL (crude drug equivalent).

2. Preparation of YCS

Separate rats were randomly divided into a YGJ-treated group and a control group (*n* = 10 per group) for the preparation of YCS and BS. The YGJ group received YGJ decoction by gavage at a dose of 5.85 g/kg once daily for 7 consecutive days, while the control group received an equal volume of saline.

One hour after the final administration, rats were deeply anesthetized with pentobarbital sodium, and terminal blood collection was performed via the abdominal aorta. Death was confirmed after exsanguination. Serum was obtained by centrifugation at 3000 rpm for 15 min at 4° C, heat-inactivated at 56° C for 30 min, and filtered through a 0.22 μm membrane. Serum samples from rats in the same group were pooled, aliquoted, and stored at −80° C until use. Serum collected from YGJ-treated rats was defined as Yiguanjian-containing serum (YCS), whereas serum collected from control rats was defined as blank serum (BS). YCS and BS were used in subsequent cell-based assays to evaluate the biological effects of serum-associated factors following YGJ administration while maintaining a matched serum background.

#### 2.2.2 UHPLC-Q-Orbitrap HRMS analysis

YGJ decoction, YCS, and BS samples were analyzed using a Vanquish ultra-high-performance liquid chromatography (UHPLC) system coupled with an Orbitrap Exploris 120 high-resolution mass spectrometer (Thermo Fisher Scientific, Waltham, MA, USA). Chromatographic separation was performed on a Phenomenex Kinetex C18 column (2.1 mm × 100 mm, 2.6 μm) maintained at 25° C. The mobile phase consisted of 0.01% acetic acid in water (A) and isopropanol/acetonitrile (1:1, v/v) (B), and the flow rate was 0.3 mL/min. The autosampler temperature was maintained at 4° C, and the injection volume was 2 μL.

MS/MS data were acquired using an electrospray ionization source in both positive and negative ion modes under the control of Xcalibur software version 4.4. Data were collected in information-dependent acquisition mode. The ESI source parameters were as follows: sheath gas flow rate, 50 Arb; auxiliary gas flow rate, 15 Arb; capillary temperature, 320° C; spray voltage, 3.8 kV in positive ion mode and −3.4 kV in negative ion mode. The full MS scan range was set to m/z 70-1,200. The full MS resolution was set to 60,000, and the MS/MS resolution was set to 15,000. The stepped normalized collision energy was set to 20/30/40.

Raw data were converted to mzXML format using ProteoWizard and processed using an in-house R program based on XCMS for peak detection, extraction, alignment, and integration. Candidate annotations were assigned to LC–MS features using BiotreeDB version 3.0 and traditional Chinese medicine-related databases (9). Candidate annotations were classified according to the detection patterns of their corresponding LC–MS features in YGJ decoction, BS, and YCS. Category I comprised candidate annotations assigned to features reported as detected in YGJ decoction and YCS but not in BS and interpreted as prototype candidates. Category II comprised candidate annotations assigned to shared-serum features reported as detected in all three sample types and classified in the original analysis as enriched in YCS relative to BS. Category III comprised candidate annotations assigned to features with a YCS-only detection pattern, reported as detected in YCS but not in YGJ decoction or BS. The Category III pattern may reflect post-administration metabolites, treatment-associated endogenous changes, or other candidate signals; however, their origins could not be definitively distinguished by the present untargeted analysis. Where more than one database annotation, including alternative molecular or adduct interpretations, was assigned to the same analytical feature, these annotations were grouped under a shared LC–MS feature ID.

#### 2.2.3 Isolation and activation of lacrimal gland epithelial cells and CD4^+^ T cells

Primary lacrimal gland epithelial cells were isolated from 1-month-old male SD rats under sterile conditions. For terminal tissue collection, rats were deeply anesthetized with pentobarbital sodium, followed by abdominal aortic blood collection. Death was confirmed before lacrimal gland dissection. Rats were then immersed in 75% ethanol for 3–5 min. Lacrimal glands were dissected on a clean bench, washed three times with PBS, and carefully cleared of surrounding fascia, adipose tissue, visible vessels, and fibrous connective tissues under a dissecting microscope. The tissues were minced into 1–2 mm fragments and digested with 0.2% type II collagenase at 37° C for approximately 25 min (10). Digestion was terminated with complete medium, and the glandular cell clusters were gently dispersed into small clusters or a single-cell suspension. The suspension was passed through a 40 μm cell strainer and centrifuged at 300 × g for 5 min. The cell pellet was resuspended in DMEM/F-12 GlutaMAX™ medium (Gibco, Grand Island, NY, USA; Cat. No. 10565018) supplemented with 20% FBS (Gibco; Cat. No. 10099-141), 20 ng/mL EGF (MedChemExpress, USA; Cat. No. HY-P7092), and antibiotics. To enrich epithelial cells, differential adhesion was performed twice for 30 min each. The collected cells were seeded on Matrigel-coated plates (Corning, USA; Cat. No. 356234) and cultured at 37° C in 5% CO_2_ until they reached an appropriate density for identification and subsequent experiments.

Splenic lymphocytes were isolated from rat spleens using a rat spleen lymphocyte separation kit according to the manufacturer's instructions. CD4⁺ T cells were purified using rat CD4 MicroBeads, and CD4⁺ T-cell enrichment was verified by flow cytometry before downstream experiments.

For T-cell activation, 24-well plates were coated with anti-rat CD3 antibody at 10 μg/mL, 400 μL per well, at 4° C for 2 h. Purified CD4⁺ T cells were then cultured in RPMI-1640 medium supplemented with 10% FBS, 1% penicillin–streptomycin, anti-rat CD28 antibody at 10 μg/mL, and recombinant rat IL-2 at 10 ng/mL. Cells were stimulated for 24 h before use in co-culture experiments.

#### 2.2.4 Identification of lacrimal gland epithelial cells

Lacrimal gland epithelial cells were identified by immunofluorescence staining for pan-cytokeratin. Cells were fixed with 4% paraformaldehyde, blocked with 5% bovine serum albumin (BSA), and incubated overnight at 4° C with anti-pan cytokeratin antibody (Bioss, Beijing, China; Cat. No. bs-5352R; 1:100). After washing, cells were incubated with Cy3-conjugated goat anti-rabbit IgG secondary antibody (ABclonal, Wuhan, China; Cat. No. AS007; 1:200). Nuclei were stained with 4′,6-diamidino-2-phenylindole (DAPI), and images were captured using a fluorescence microscope.

#### 2.2.5 Screening of optimal intervention concentrations

Cell viability was assessed using a Cell Counting Kit-8 (CCK-8) assay (KeyGEN BioTECH, China; Cat. No. KGA9305-500). Lacrimal gland epithelial cells and activated CD4⁺ T cells were treated with YCS or BS at final concentrations of 0%, 1%, 3%, 5%, 10%, and 20% for 24 h. For lacrimal gland epithelial cells, 10 μL of CCK-8 reagent was added to each well and incubated for 2 h before absorbance was measured at 450 nm. For activated CD4⁺ T cells, 10 μL of CCK-8 reagent was added to each well and incubated for 4 h before absorbance measurement at 450 nm. In parallel, CD4⁺ T cells from the Control, 5% BS, 10% BS, 5% YCS, and 10% YCS groups were collected for flow cytometric assessment of Treg cell differentiation. Based on these screening results, 5% YCS and 5% BS were selected for subsequent experiments.

Activated CD4⁺ T cells were further treated with AS2863619 (MedChemExpress; Cat. No. HY-126675A) or Peptide P60 (MedChemExpress; Cat. No. HY-P1684) for concentration screening. For the viability assay, cells were treated with AS2863619 at 0, 10, 100, 500, 1,000, and 2,000 nM or with Peptide P60 at 0, 0.01, 0.05, 0.1, 1, and 10 μM for 24 h. CCK-8 reagent was then added at 10 μL per well, followed by incubation for 4 h and absorbance measurement at 450 nm. To further evaluate their effects on Treg cell differentiation, CD4⁺ T cells assigned to AS2863619 screening concentrations of 0, 0.5, and 1 μM or Peptide P60 screening concentrations of 0, 1, and 10 μM were collected for flow cytometric analysis. Based on these screening experiments, 0.5 μM AS2863619 and 10 μM Peptide P60 were selected for subsequent co-culture experiments.

#### 2.2.6 Establishment of the hyperosmotic epithelial injury model

To establish the *in vitro* hyperosmotic stress condition, lacrimal gland epithelial cells were exposed to culture medium supplemented with 70, 90, or 120 mM NaCl for 24 h (11). Cells maintained under normal osmotic conditions were used as the Control group. Cell viability was assessed using the CCK-8 assay, and IL-17A mRNA expression was evaluated by RT-qPCR. Based on the combined changes in epithelial cell viability and inflammatory gene expression, 120 mM NaCl for 24 h was selected for subsequent co-culture experiments.

#### 2.2.7 Co-culture system and experimental design

A non-contact co-culture system was established using 0.4 μm Transwell inserts. Lacrimal gland epithelial cells were seeded in the upper chamber, and activated CD4⁺ T cells were seeded in the lower chamber at a 5:1 epithelial cell-to-CD4⁺ T-cell ratio. CD4⁺ T cells were activated for 24 h before co-culture. After the upper chamber containing lacrimal gland epithelial cells was transferred into the co-culture plate, activated CD4⁺ T cells were added to the lower chamber, and the indicated interventions were applied for 24 h. The Transwell system allowed the exchange of soluble mediators between the two cell populations without direct cell–cell contact.

Co-culture was performed in RPMI-1640 medium supplemented with 10% FBS and 1% penicillin–streptomycin, together with the indicated serum or pharmacological intervention. Hyperosmotic co-culture conditions were established by adding 120 mM NaCl for 24 h. YCS, BS, AS2863619, and Peptide P60 were added according to the experimental design at the beginning of co-culture. YCS and BS were used at a final concentration of 5%, whereas AS2863619 and Peptide P60 were used at final concentrations of 0.5 μM and 10 μM, respectively.

Two independent co-culture intervention sets were performed to evaluate YCS alone or in combination with pharmacological modulators of Foxp3-associated regulation. In the first set, cells were assigned to the Control, Model, Model + YCS, and Model + AS2863619 groups. In the second set, cells were assigned to the Model, Model + YCS, Model + Peptide P60, and Model + YCS + Peptide P60 groups. The Control group was maintained under normal osmotic conditions and received 5% BS. The Model group was exposed to 120 mM NaCl and received 5% BS. The Model + YCS group received 5% YCS under hyperosmotic conditions. The Model + AS2863619 group received 5% BS and 0.5 μM AS2863619 under hyperosmotic conditions. The Model + Peptide P60 group received 5% BS and 10 μM Peptide P60 under hyperosmotic conditions. The Model + YCS + Peptide P60 group received 5% YCS and 10 μM Peptide P60 under hyperosmotic conditions.

After 24 h of co-culture, lacrimal gland epithelial cells from the upper chamber and CD4⁺ T cells from the lower chamber were collected for subsequent analyses.

#### 2.2.8 Measurement of intracellular ROS

Intracellular ROS levels in CD4⁺ T cells collected from the lower chamber were measured using a Reactive Oxygen Species Assay Kit (KeyGEN BioTECH; Cat. No. KGT010-1). After co-culture, CD4⁺ T cells from the lower chamber were collected and incubated with DCFH-DA diluted 1:1,000 in serum-free medium to a final concentration of 10 μmol/L. Cells were incubated at 37° C for 20 min and then washed three times with serum-free medium to remove extracellular DCFH-DA. After an additional wash with 1 mL PBS, cells were centrifuged at 1,500 rpm for 5 min, resuspended in 300 μL PBS, and analyzed using a NovoCyte 2060R flow cytometer. Intracellular ROS levels were quantified according to DCFH-DA fluorescence intensity (12).

#### 2.2.9 Flow cytometric analysis of Th17 and Treg cells

After co-culture, CD4⁺ T cells from the lower chamber were collected for flow cytometric analysis of T helper 17 (Th17) and regulatory T cells (Treg). For Th17 analysis, cells were stimulated with phorbol 12-myristate 13-acetate (PMA; 50 ng/mL; MedChemExpress; Cat. No. HY-18739), ionomycin (1 μg/mL; MedChemExpress; Cat. No. HY-13434), and brefeldin A (BFA; 5 μg/mL; MedChemExpress; Cat. No. HY-16592) for 4 h before staining (13). Cells were then stained with APC-conjugated anti-CD4 antibody (BioLegend; Cat. No. 201509; 1:100), followed by fixation, permeabilization, and intracellular staining with phycoerythrin (PE)-conjugated anti-interleukin-17A (IL-17A) antibody (Invitrogen; Cat. No. 12-7177-81; 1:100).

For Treg analysis, cells were stained with allophycocyanin (APC)-conjugated anti-CD4 antibody (BioLegend; Cat. No. 201509; 1:100) and FITC-conjugated anti-CD25 antibody (BioLegend; Cat. No. 202103; 1:100), followed by fixation, permeabilization, and intracellular staining with PE-conjugated anti-Foxp3 antibody (BioLegend; Cat. No. 320008; 1:100).

Data acquisition was performed using a NovoCyte 2060R flow cytometer, and data were analyzed using NovoExpress software version 1.5.0.

#### 2.2.10 Immunofluorescence staining

CD4⁺ T cells were fixed and blocked before immunofluorescence staining. For Foxp3 staining, cells were permeabilized and then incubated with primary antibody against Foxp3 (Bioss; Cat. No. bs-10211R; 1:200) overnight at 4° C. For IL-17A staining, cells were incubated with primary antibody against IL-17A (Affinity Biosciences; Cat. No. DF6127; 1:200). Triton X-100 permeabilization was not performed for IL-17A staining according to the experimental protocol. After washing, cells were incubated with Cy3-conjugated secondary antibody (ABclonal; Cat. No. AS007; 1:200).

Nuclei were counterstained with 4′,6-diamidino-2-phenylindole (DAPI), and fluorescence images were acquired using a BX53 fluorescence microscope (Olympus, Tokyo, Japan). For quantitative analysis, three independent experiments were performed for each group. One slide from each independent experiment was analyzed, and three randomly selected fields were quantified per slide. The mean of the three fields from each slide was used as one independent experimental value for statistical analysis (*n* = 3). These three independent experimental values are displayed in Figures 11, 12. Fluorescence intensity was quantified using ImageJ and expressed as integrated optical density per area (IOD/Area).

**Figure 1:**
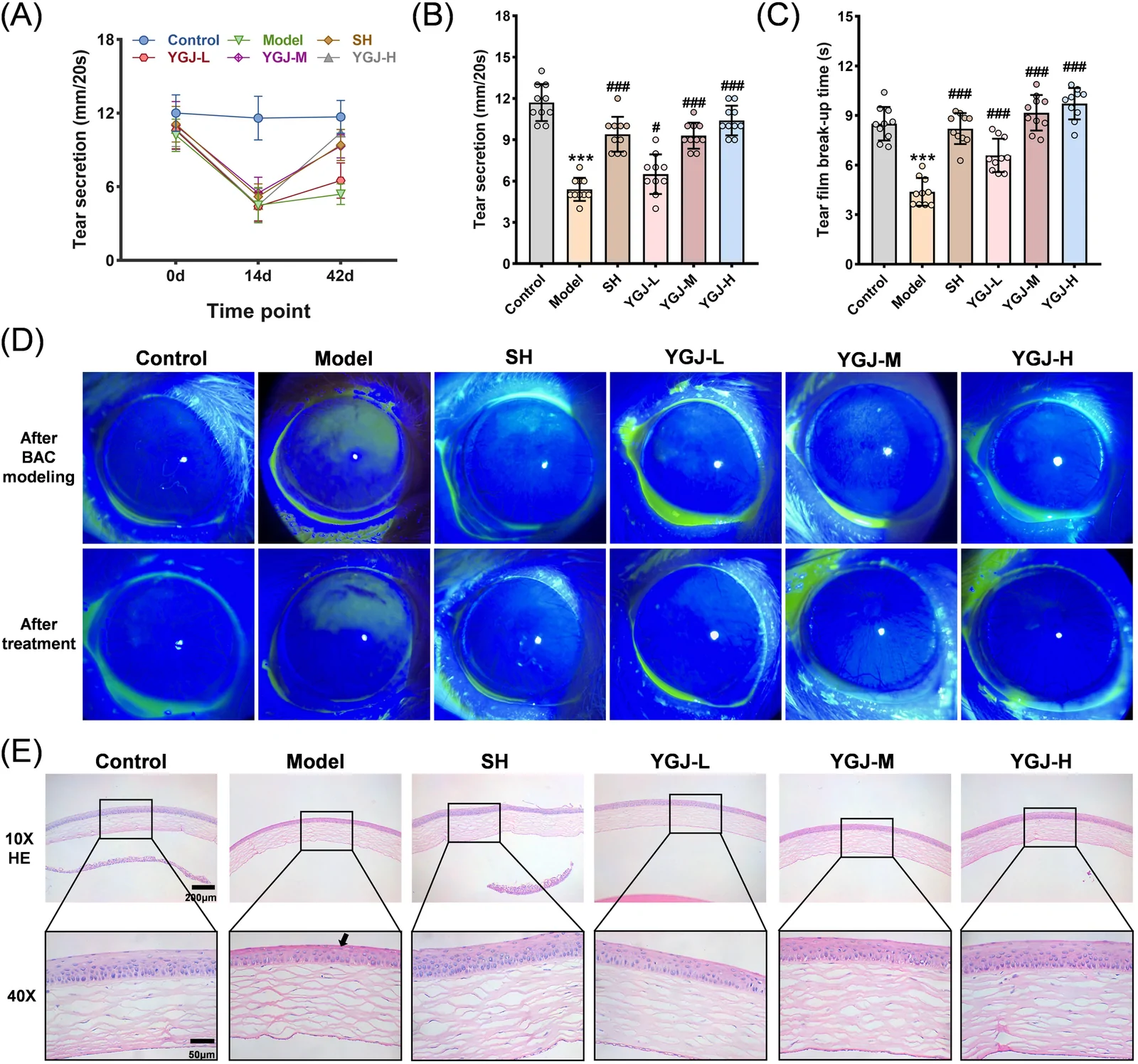
YGJ improved tear secretion and tear film stability and reduced corneal epithelial injury in BAC-induced dry eye rats. **(A)** Tear secretion was dynamically measured using phenol red thread before BAC modeling, after BAC modeling, and after treatment. **(B)** Quantitative analysis of tear secretion after treatment. **(C)** Quantitative analysis of tear film break-up time after treatment. **(D)** Representative corneal fluorescein staining images showing corneal epithelial injury. **(E)** Representative HE staining images of corneal tissues showing epithelial morphology in each group. In the Model group, the black arrow indicates representative corneal epithelial disorganization. Data are presented as mean ± SD. For panels **(A–C)**, *n* = 10 rats per group. Representative images are shown for panels **(D,E)**. Statistical significance was determined by one-way ANOVA followed by appropriate *post hoc* tests. *P < 0.05, **P < 0.01, ***P < 0.001 vs. the Control group; ^#^*P* < 0.05, ^##^*P* < 0.01, ^###^*P* < 0.001 vs. the Model group.

#### 2.2.11 Reverse transcription quantitative PCR (RT-qPCR) analysis

Total RNA was extracted using TRIzol reagent (CWBIO, Beijing, China; Cat. No. CW0580S) and purified using an RNA extraction kit (CWBIO; Cat. No. CW0581M). RNA concentration was measured using a NanoPhotometer NP80 spectrophotometer.

cDNA was synthesized using HiScript II Q RT SuperMix (Vazyme, China; Cat. No. R223-01). Reverse transcription quantitative PCR (RT-qPCR) was performed using SYBR Master Mix (CWBIO; Cat. No. CW3360M) on a CFX Connect™ Real-Time PCR System (Bio-Rad, USA). Three independent experiments were performed for each group, and each experiment was measured in technical triplicate. The mean of the technical triplicates was used as one value for the corresponding independent experiment in the statistical analysis. Relative gene expression was calculated using the 2^∧^−ΔΔCt method with GAPDH as the internal control (14).

In CD4⁺ T cells, the expression of Th17-related genes, including RORγt, IL-17A, and interferon-gamma (IFN-γ), and Treg-related genes, including Foxp3, transforming growth factor-beta (TGF-β), and interleukin-10 (IL-10), was examined. In lacrimal gland epithelial cells, IL-17A, TGF-β, and IL-10 expression was also detected. Primer sequences for the *in vitro* cell experiments are provided in Supplementary Table S2.

### 2.3 Statistical analysis

Statistical analyses were performed using SPSS software version 26.0. Graphs were generated using GraphPad Prism software version 8.0. Data are presented as mean ± standard deviation (SD). The independent experimental unit and sample size for each assay are specified in the corresponding figure legends. For the animal-tissue analyses shown in Figures 3, 4, *n* denotes the number of independent rats. For the primary-cell assays, *n* denotes the number of independently performed experiments. Technical wells, imaging fields, technical PCR replicates, or repeated measurements within the same experiment were averaged as appropriate and were not treated as independent observations. Normality was assessed for datasets with more than three independent observations per group. Formal group-wise normality testing was not performed for datasets with *n* = 3 because of its limited diagnostic value at this sample size. Homogeneity of variance was assessed for all group comparisons.

For comparisons among multiple groups, one-way analysis of variance (ANOVA) was used. When the assumption of homogeneity of variance was met, the least significant difference (LSD) test was used for *post hoc* comparisons; otherwise, Dunnett's T3 test was applied. For the preliminary serum concentration screening assays comparing YCS with BS at the same concentration, comparisons were performed using an unpaired two-tailed Student's *t*-test. A value of *P* < 0.05 was considered statistically significant.

## 3 Results

### 3.1 YGJ alleviated ocular surface injury and lacrimal gland immune dysregulation in BAC-induced dry eye rats

#### 3.1.1 YGJ improved tear secretion and tear film stability and reduced corneal epithelial injury in BAC-induced dry eye rats

Tear secretion was monitored before BAC modeling, after BAC modeling, and after treatment. Before BAC exposure, tear secretion was comparable among groups. After BAC modeling, tear secretion was markedly reduced in the model and treatment-designated groups compared with the Control group, confirming the successful induction of a DED phenotype. After treatment, tear secretion remained reduced in the Model group, whereas SH and YGJ increased tear secretion to varying degrees. This improvement was more evident in the SH, YGJ-M, and YGJ-H groups than in the YGJ-L group (Figures 1A,B).

Tear film break-up time was assessed to evaluate tear film stability. After treatment, the Model group continued to show tear film instability, whereas SH and YGJ treatment prolonged tear film break-up time, with more pronounced recovery in the YGJ-M and YGJ-H groups (Figure 1C).

Corneal fluorescein staining revealed increased fluorescein-positive areas in the Model group, indicating corneal epithelial barrier disruption. After treatment, fluorescein staining was reduced in the SH and YGJ-treated groups, particularly in the YGJ-M and YGJ-H groups (Figure 1D). HE staining further showed a relatively regular corneal epithelial structure in the Control group, whereas BAC exposure caused epithelial disorganization, impaired stratification, reduced surface regularity, and focal vacuolar-like changes. SH and YGJ treatment partially restored corneal epithelial morphology (Figure 1E).

#### 3.1.2 YGJ alleviated lacrimal gland histopathological injury and partially preserved meibomian gland and conjunctival morphology

HE staining showed well-preserved lacrimal gland acini with compact arrangement and limited stromal space in the Control group. In the Model group, BAC exposure induced evident lacrimal gland injury, characterized by disorganized acinar architecture, widened inter-acinar spaces, and disrupted glandular structure. These changes were accompanied by focal accumulations of densely stained small nuclei in the interstitial regions, suggestive of inflammatory-cell infiltration. Compared with the Model group, SH and YGJ treatment showed partial improvement in lacrimal gland morphology, with more regular acinar arrangement and less prominent stromal expansion across the treatment groups (Figure 2A).

**Figure 2:**
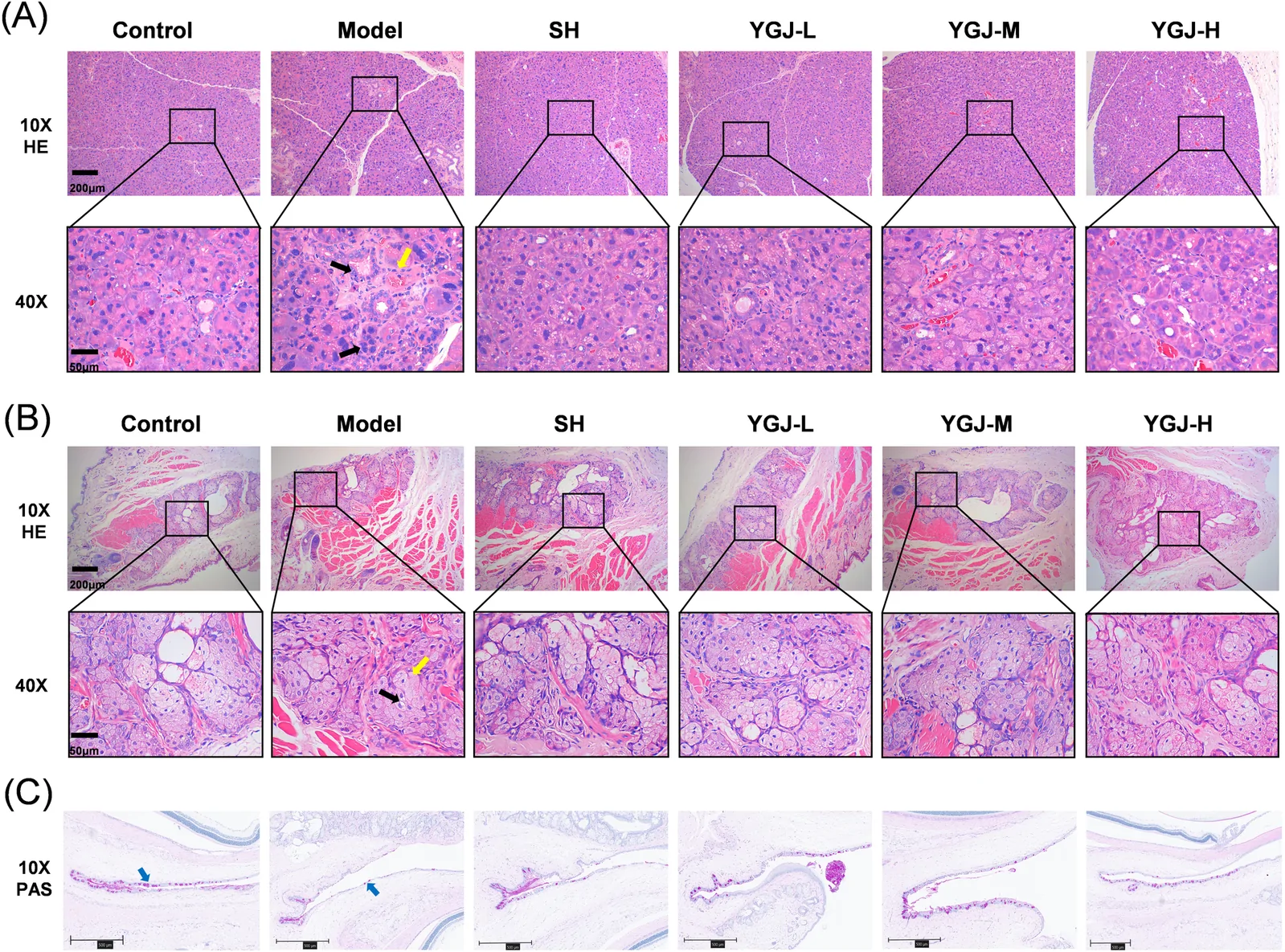
YGJ alleviated lacrimal gland histopathological injury and partially preserved meibomian gland and conjunctival morphology in BAC-induced dry eye rats. (**A**) Representative HE staining images of lacrimal gland tissues showing acinar architecture and interstitial changes. In the Model group, black arrows indicate focal accumulations of densely stained small interstitial nuclei, suggestive of inflammatory-cell infiltration, whereas the yellow arrow indicates a representative widened inter-acinar space. (**B**) Representative HE staining images of meibomian gland tissues showing glandular morphology and pale vacuolated areas. In the Model group, the black arrow indicates a representative pale vacuolated area, whereas the yellow arrow indicates relatively irregular glandular morphology. (**C**) Representative PAS staining images of conjunctival tissues. Blue arrows indicate representative PAS-positive regions along the conjunctival epithelium. Representative images are shown for all panels.

In meibomian gland tissues, the Control group showed recognizable glandular structures with pale vacuolated areas. Compared with the Control group, the Model group showed relatively irregular glandular morphology and a more uneven distribution of pale vacuolated areas. SH and YGJ-treated groups showed partially preserved glandular morphology; however, these changes were less prominent than those observed in lacrimal gland tissues (Figure 2B).

PAS staining was used to evaluate PAS-positive mucin-associated regions along the conjunctival epithelium. Compared with the Control group, the Model group showed reduced and less continuous PAS-positive staining along the conjunctival epithelium. SH and YGJ treatment showed partial recovery of PAS-positive staining to varying degrees, suggesting a tendency toward improved mucin-associated epithelial preservation (Figure 2C).

#### 3.1.3 YGJ treatment was associated with partial restoration of the lacrimal gland Th17/Treg balance in BAC-induced dry eye rats

To determine whether YGJ modulated local immune dysregulation in BAC-induced dry eye rats, Th17 and Treg cells in lacrimal gland tissues were analyzed by flow cytometry. The proportion of Th17 cells was increased in the Model group compared with the Control group, indicating enhanced local pro-inflammatory T cell differentiation after BAC-induced dry eye modeling. Compared with the Model group, SH and YGJ treatment reduced the proportion of Th17 cells, with a more evident inhibitory effect in the YGJ-M and YGJ-H groups (Figure 3A).

**Figure 3:**
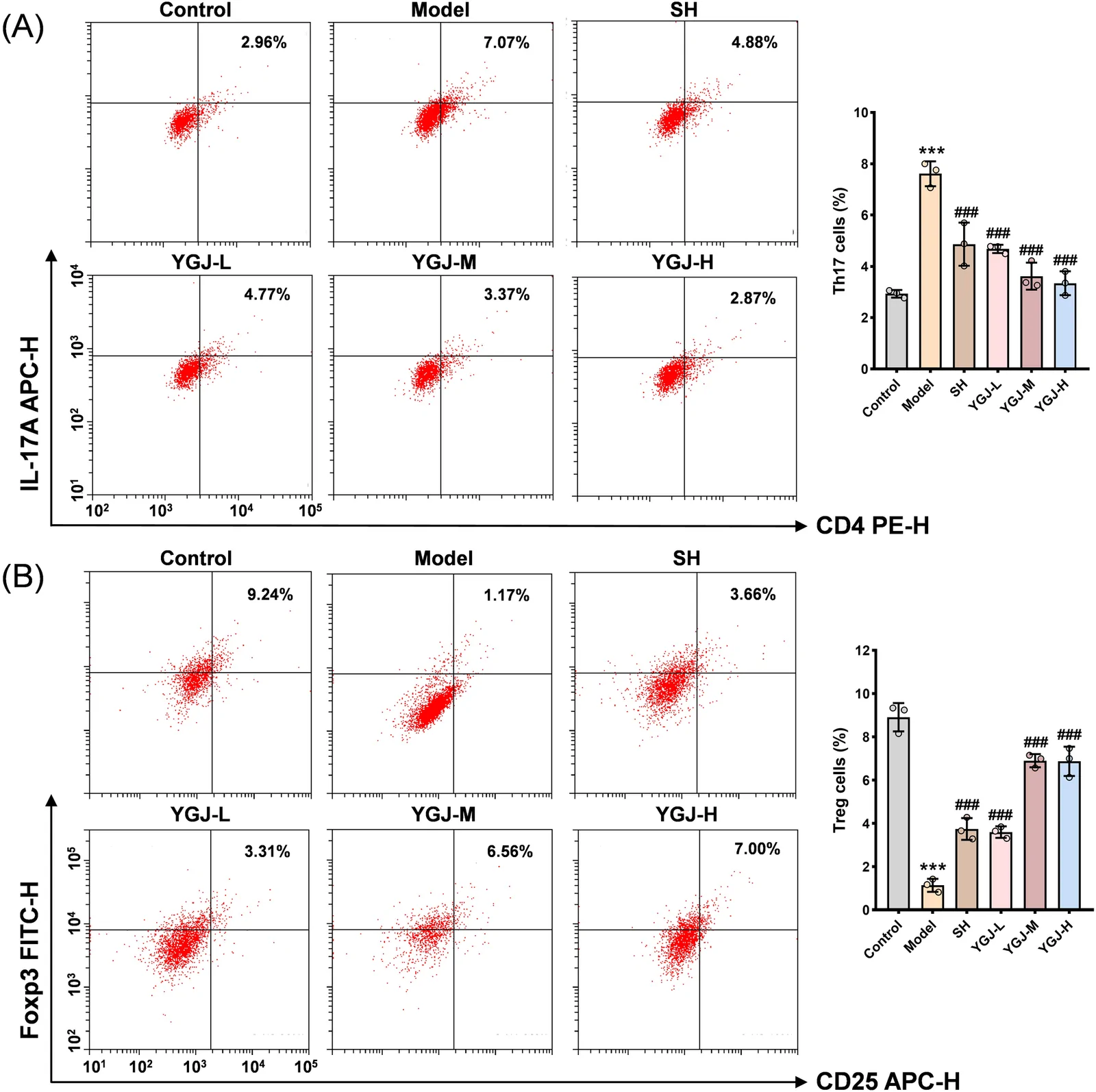
YGJ treatment was associated with partial restoration of the lacrimal gland Th17/Treg balance in BAC-induced dry eye rats. Flow cytometry was performed to evaluate Th17 and Treg cell proportions in lacrimal gland tissues. **(A)** Representative flow cytometry plots and quantitative analysis of CD4⁺IL-17A⁺ Th17 cells. **(B)** Representative flow cytometry plots and quantitative analysis of CD4⁺CD25⁺Foxp3⁺ Treg cells. Only the percentage of the upper-right target population is displayed in the representative plots. Data are presented as mean ± SD. For panels A and B, *n* = 3 independent rats per group. Statistical significance was determined by one-way ANOVA followed by appropriate *post hoc* tests. *P < 0.05, ^**^P < 0.01, ***P < 0.001 vs. the Control group; ^#^*P* < 0.05, ^##^*P* < 0.01, ^###^*P* < 0.001 vs. the Model group.

In contrast, the proportion of Treg cells was decreased in the Model group compared with the Control group, suggesting impaired local immunoregulatory capacity. SH and YGJ treatment increased the proportion of Treg cells, with more pronounced restoration in the YGJ-M and YGJ-H groups (Figure 3B). Together, these findings were consistent with a YGJ-associated shift from Th17 predominance toward Treg-associated immunoregulation in the lacrimal gland.

#### 3.1.4 YGJ treatment was associated with changes in Th17/Treg-related markers and reduced STAT3 phosphorylation in lacrimal gland tissues

To further characterize the molecular changes associated with lacrimal gland immune regulation, Th17/Treg-related markers and STAT3 signaling were examined in lacrimal gland tissues. RT-qPCR analysis showed that BAC-induced dry eye reduced the expression of the Treg-related genes Foxp3 and IL-10, while increasing the expression of the Th17-related genes RORγt and IL-17A in the Model group. SH and YGJ treatment reversed these transcriptional changes to varying degrees, as reflected by increased Foxp3 and IL-10 expression and decreased RORγt and IL-17A expression. The regulatory effect was more apparent in the YGJ-M and YGJ-H groups (Figures 4A–D).

**Figure 4:**
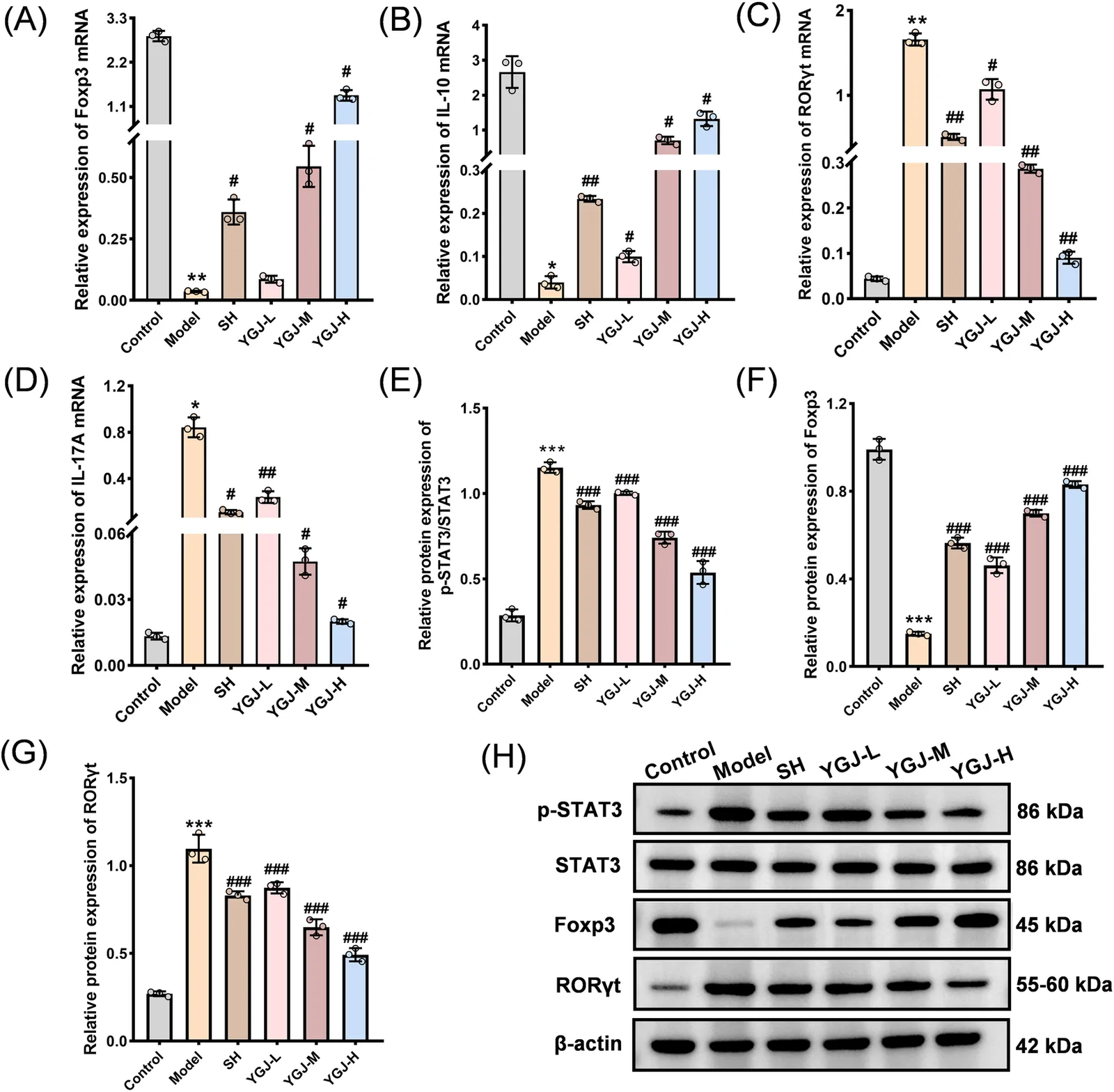
YGJ treatment was associated with changes in Th17/Treg-related markers and reduced STAT3 phosphorylation in lacrimal gland tissues. **(A–D)** RT-qPCR analysis of Foxp3, IL-10, RORγt, and IL-17A mRNA expression in lacrimal gland tissues. **(E–G)** Quantitative analysis of p-STAT3/STAT3, Foxp3, and RORγt protein expression. **(H)** Representative western blot bands of p-STAT3, STAT3, Foxp3, RORγt, and β-actin in lacrimal gland tissues. Data are presented as mean ± SD. For each assay in panels **(A–G)**, *n* = 3 independent rats per group. Panel **(H)** shows representative western blot bands. Statistical significance was determined by one-way ANOVA followed by appropriate *post hoc* tests. ^*^P < 0.05, **P < 0.01, ***P < 0.001 vs. the Control group; ^#^*P* < 0.05, ^##^*P* < 0.01, ^###^*P* < 0.001 vs. the Model group.

Western blot analysis showed an increased p-STAT3/STAT3 ratio in lacrimal gland tissues from the Model group, indicating STAT3 activation after BAC-induced dry eye modeling. SH and YGJ treatment reduced the p-STAT3/STAT3 ratio, accompanied by increased Foxp3 protein expression and decreased RORγt protein expression (Figures 4E–G). Representative western blot bands are shown in Figure 4H. These molecular changes were consistent with partial normalization of Th17/Treg-related markers and reduced STAT3 phosphorylation after YGJ treatment.

### 3.2 Serum pharmacochemistry and *in vitro* epithelial–immune co-culture validation

#### 3.2.1 UHPLC-Q-Orbitrap HRMS characterized the circulating chemical profile of YCS

The replicate total ion chromatograms (TICs) of YGJ decoction and YCS in both negative and positive ion modes showed consistent chromatographic profiles, indicating good analytical stability and reproducibility of the LC–MS/MS analysis (Supplementary Figure S1). Based on MS/MS spectral matching and database searching, the LC–MS features detected in YGJ, BS, and YCS were assigned candidate annotations (Supplementary Table S3).

Serum pharmacochemistry yielded 67 candidate annotations corresponding to 56 distinct LC–MS features in YCS. Based on the detection-pattern classification provided in the original serum pharmacochemistry report, these annotations comprised 6 category I annotations corresponding to 6 prototype candidate features, 40 category II annotations corresponding to 29 YCS-enriched shared-serum features, and 21 category III annotations corresponding to 21 features with a YCS-only detection pattern (Supplementary Table S4). Category III represents an analytical detection pattern rather than confirmation that these features are YGJ-derived metabolites. Structural classification showed that the annotations included shikimates and phenylpropanoids (*n* = 23), alkaloids (*n* = 10), terpenoids (*n* = 10), fatty acids (*n* = 8), amino acids and peptides (*n* = 5), carbohydrates (*n* = 4), and polyketides (*n* = 2). For five annotations, no chemical superclass was available in the original annotation output. Where more than one candidate structure was assigned to the same LC–MS feature, these entries were retained as alternative annotations rather than treated as independently confirmed compounds. Representative TICs of YGJ decoction and YCS in positive and negative ion modes are shown in Figures 5, 6, respectively. Assignments of the numbered peaks in these chromatograms are provided in Supplementary Table S5. Together, these findings demonstrate a chemically diverse circulating profile after YGJ administration and provide a serum pharmacochemical basis for the subsequent epithelial–immune co-culture experiments.

**Figure 5:**
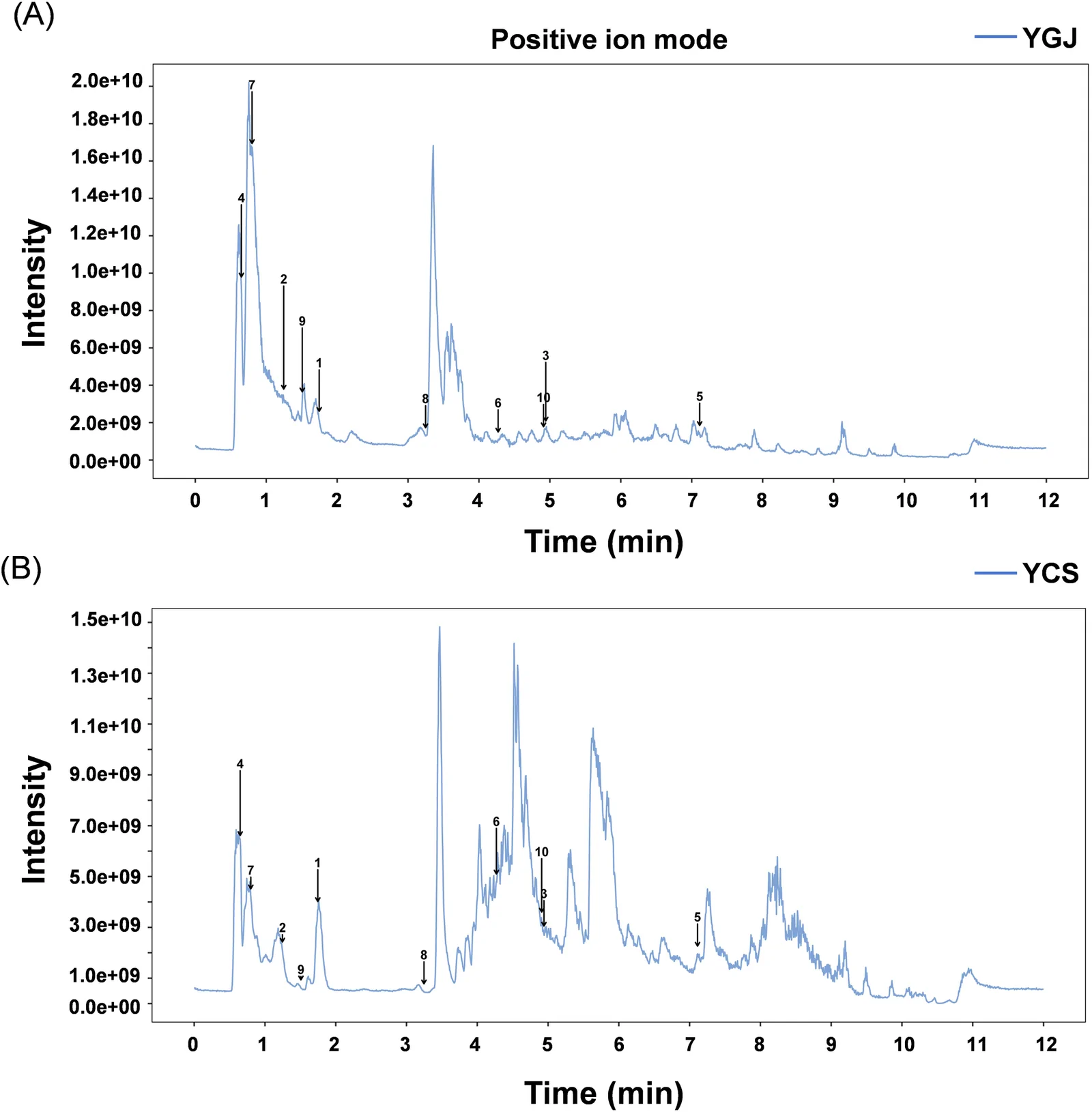
Representative TICs of YGJ decoction and YCS in positive ion mode. **(A)** Representative total ion chromatogram (TIC) of YGJ decoction. **(B)** Representative TIC of YCS. Numbered peaks denote chromatographic peaks with candidate annotations; their assignments are provided in Supplementary Table S5.

**Figure 6:**
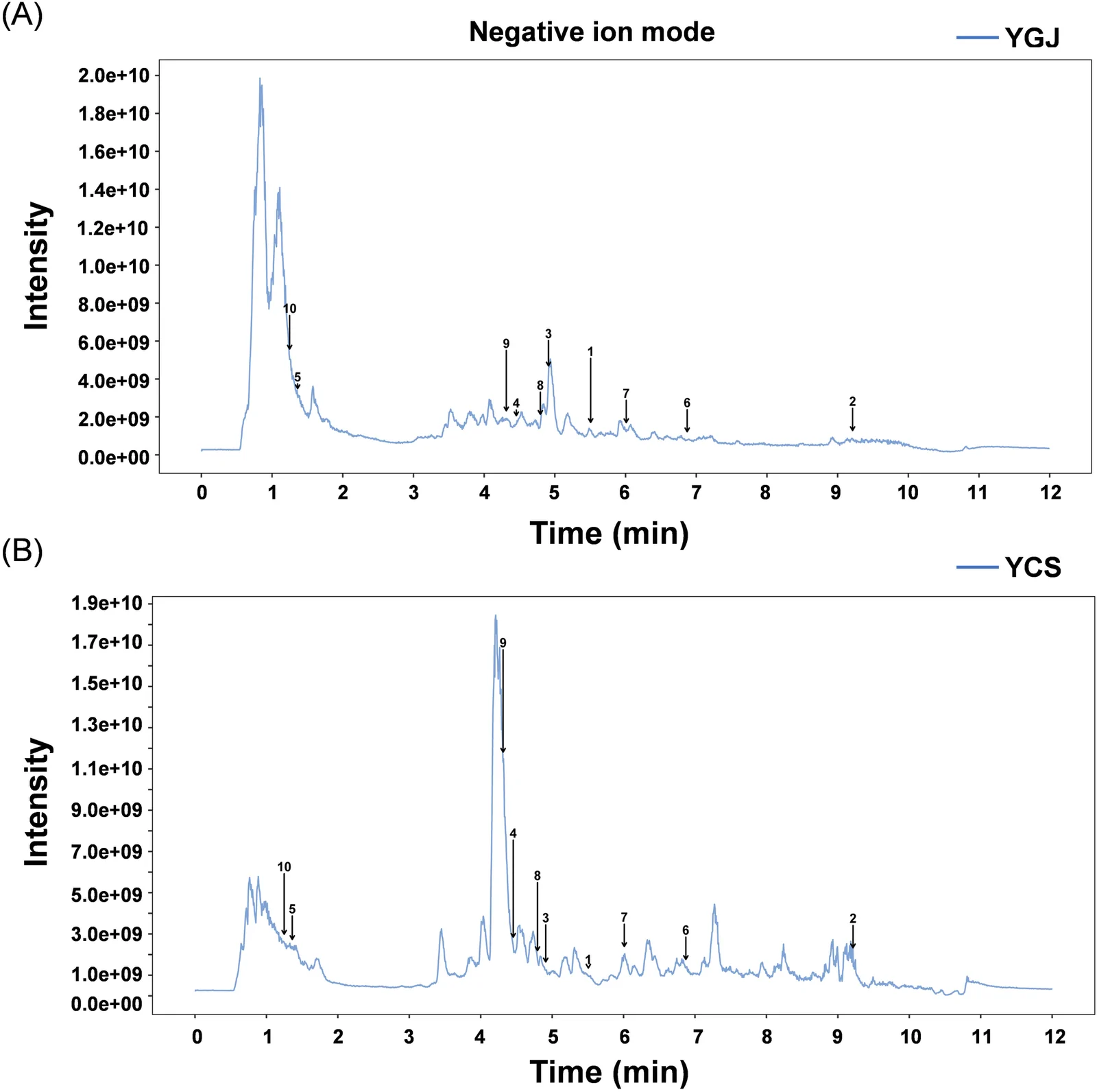
Representative TICs of YGJ decoction and YCS in negative ion mode. **(A)** Representative total ion chromatogram (TIC) of YGJ decoction. **(B)** Representative TIC of YCS. Numbered peaks denote chromatographic peaks with candidate annotations; their assignments are provided in Supplementary Table S5.

#### 3.2.2 Preparation and identification of lacrimal gland epithelial cells and CD4^+^ T cells for co-culture experiments

Primary lacrimal gland epithelial cells were successfully isolated from rat lacrimal gland tissues and cultured *in vitro*. After attachment, the cells gradually displayed an epithelial-like morphology, with a polygonal shape and cobblestone-like arrangement. Immunofluorescence staining showed strong cytoplasmic pan-cytokeratin positivity with clear DAPI nuclear staining, confirming the epithelial phenotype of the isolated cells (Figure 7A).

**Figure 7:**
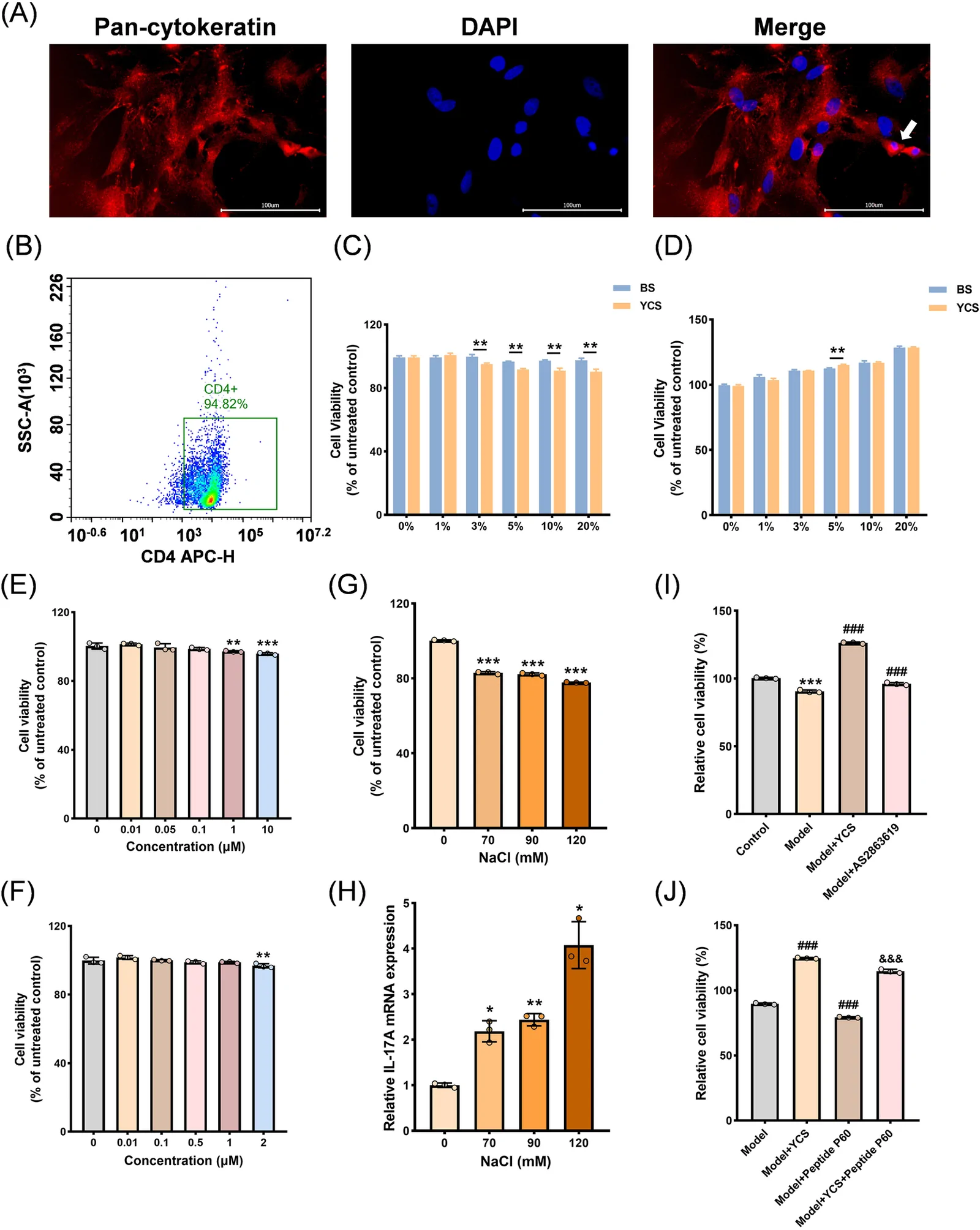
Identification of primary lacrimal gland epithelial cells and CD4⁺ T cells, selection of intervention conditions, and evaluation of lacrimal gland epithelial cell viability. **(A)** Immunofluorescence staining of primary lacrimal gland epithelial cells for pan-cytokeratin. Red fluorescence indicates pan-cytokeratin staining, and blue fluorescence indicates DAPI-stained nuclei. The white arrow in the merged image indicates representative pan-cytokeratin-positive cytoplasmic staining in a lacrimal gland epithelial cell. Scale bar = 100 μm. **(B)** Flow cytometric analysis showing the purity of CD4⁺ T cells after magnetic bead sorting. **(C)** Effects of different concentrations of BS and YCS on CD4⁺ T-cell viability. **(D)** Effects of different concentrations of BS and YCS on lacrimal gland epithelial cell viability. **(E)** Effects of different concentrations of Peptide P60 on CD4⁺ T-cell viability. **(F)** Effects of different concentrations of AS2863619 on CD4⁺ T-cell viability. **(G)** Effects of different NaCl concentrations on lacrimal gland epithelial cell viability after 24 h of treatment. (**H**) Effects of different NaCl concentrations on IL-17A mRNA expression in lacrimal gland epithelial cells. **(I)** Effects of YCS and AS2863619 on lacrimal gland epithelial cell viability under hyperosmotic co-culture conditions. **(J)** Effects of YCS and Peptide P60 on lacrimal gland epithelial cell viability under hyperosmotic co-culture conditions. Cell viability was evaluated using the CCK-8 assay. Data are presented as mean ± SD. For panels **(C–J)**, *n* = 3 independent experiments per group or condition. Statistical significance for panels **(C,D)** was determined using an unpaired two-tailed Student’s *t*-test between YCS and the corresponding BS group at the same concentration; *P < 0.05, **P < 0.01 vs. the corresponding BS group. For panels **(E–J)**, statistical significance was determined by one-way ANOVA followed by appropriate *post hoc* tests. For panels E and F, **P < 0.01, ***P < 0.001 vs. the untreated group. For panels G and H, *P < 0.05, **P < 0.01, ***P < 0.001 vs. the 0 mM group. For panel I, ^***^P < 0.001 vs. the Control group; ^###^*P* < 0.001 vs. the Model group. For panel J, ^###^*P* < 0.001 vs. the Model group; ^&&&^*P* < 0.001 vs. the Model + Peptide P60 group.

CD4⁺ T cells were isolated from rat spleens and purified by magnetic bead sorting. Flow cytometric analysis confirmed efficient enrichment of CD4⁺ T cells (Figure 7B). These results confirmed that primary lacrimal gland epithelial cells and CD4⁺ T cells were successfully prepared for subsequent co-culture experiments.

#### 3.2.3 Selection of YCS and pharmacological modulator concentrations for co-culture experiments

To establish appropriate intervention conditions for subsequent experiments, the effects of YCS and BS on cell viability were first evaluated using a CCK-8 assay. In CD4⁺ T cells, treatment with YCS at concentrations ranging from 0% to 20% for 24 h did not markedly reduce cell viability, although viability was significantly lower in the 3%, 5%, 10%, and 20% YCS groups than in the corresponding BS groups at the same concentrations (Figure 7C). In lacrimal gland epithelial cells, YCS was well tolerated, and 5% YCS significantly increased cell viability compared with the corresponding 5% BS group (Figure 7D). Considering its tolerability in CD4⁺ T cells, its favorable effect on lacrimal gland epithelial cell viability, and its Treg-promoting effect, 5% YCS was selected for subsequent experiments.

The effects of Peptide P60 and AS2863619 on CD4⁺ T cell viability were then assessed. Peptide P60 showed limited effects on CD4⁺ T cell viability at low concentrations, whereas higher concentrations reduced cell viability to varying degrees (Figure 7E). AS2863619 showed no obvious cytotoxicity at concentrations ≤1 μM, while 2 μM AS2863619 significantly reduced cell viability (Figure 7F).

To further evaluate the immunomodulatory effects of these interventions, Treg differentiation was assessed by flow cytometry. The proportion of Treg cells was higher in the 5% YCS group than in the 5% BS group (Figure 8A). In the Treg differentiation assay, 10 μM Peptide P60 produced a marked inhibitory effect on Treg differentiation, whereas 0.5 μM AS2863619 increased the proportion of Treg cells (Figures 8B,C). Representative flow cytometry plots further supported these changes in CD25 and Foxp3 staining among the BS, YCS, Peptide P60, and AS2863619 groups (Figure 8D).

**Figure 8:**
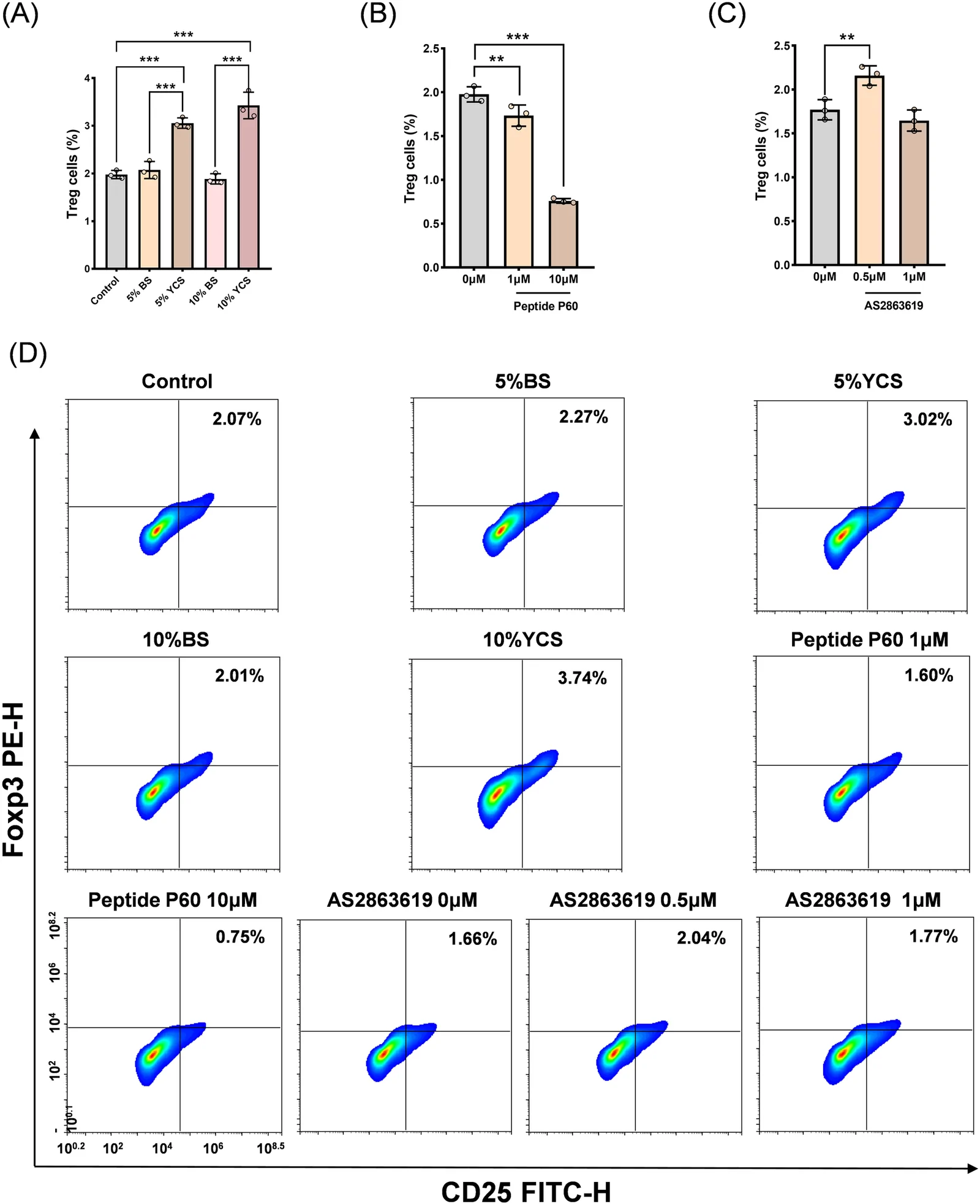
Screening of YCS and pharmacological modulators based on Treg differentiation. Treg differentiation was evaluated by flow cytometry. **(A)** Effects of different concentrations of BS and YCS on the proportion of CD4⁺CD25⁺Foxp3⁺ Treg cells. **(B)** Effects of different concentrations of Peptide P60 on Treg differentiation. **(C)** Effects of different concentrations of AS2863619 on Treg differentiation. **(D)** Representative flow cytometry plots of CD25 and Foxp3 staining in CD4⁺ T cells treated with BS, YCS, Peptide P60, or AS2863619. Data are presented as mean ± SD. For panels A–C, *n* = 3 independent experiments per group. Representative flow cytometry plots are shown for panel **(D)**. Statistical significance was determined by one-way ANOVA followed by appropriate *post hoc* tests. *P < 0.05, **P < 0.01, ^***^P < 0.001 for the indicated comparisons.

Based on the combined results of cell viability and Treg differentiation screening, 5% YCS and 5% BS were used as serum intervention conditions, 0.5 μM AS2863619 was selected as a Treg-promoting intervention, and 10 μM Peptide P60 was used as a Foxp3/Treg-disrupting intervention in subsequent mechanistic experiments.

#### 3.2.4 Establishment of a hyperosmotic lacrimal gland epithelial injury condition

To establish an *in vitro* hyperosmotic epithelial injury model, lacrimal gland epithelial cells were treated with different concentrations of NaCl for 24 h. Cell viability was evaluated using the CCK-8 assay, and IL-17A mRNA expression was detected by RT-qPCR. Compared with the 0 mM group, treatment with 70, 90, and 120 mM NaCl significantly reduced cell viability (all *P* < 0.001), with the most pronounced decrease observed in the 120 mM group (Figure 7G). Meanwhile, NaCl treatment increased IL-17A mRNA expression, with a marked elevation observed in the 120 mM group (*P* = 0.027) (Figure 7H). Considering both the decrease in epithelial cell viability and the induction of IL-17A expression, 120 mM NaCl for 24 h was selected to establish the hyperosmotic epithelial injury condition for subsequent co-culture experiments.

#### 3.2.5 YCS preserved lacrimal gland epithelial cell viability under hyperosmotic epithelial–immune co-culture conditions

After 120 mM NaCl was selected as the hyperosmotic modeling condition, the viability of lacrimal gland epithelial cells in the co-culture system was further evaluated using the CCK-8 assay. Compared with the Control group, the Model group showed a significant reduction in lacrimal gland epithelial cell viability (*P* < 0.001), indicating that hyperosmotic co-culture conditions induced epithelial cell injury. Compared with the Model group, cell viability was significantly increased in the Model + YCS group (*P* < 0.001), and a similar increase was observed in the Model + AS2863619 group (*P* < 0.001) (Figure 7I).

To further examine whether the effect of YCS was associated with Foxp3-related regulation, Peptide P60 was introduced in a separate experiment. Compared with the Model group, cell viability was significantly increased in the Model + YCS group (*P* < 0.001), whereas it was further reduced in the Model + Peptide P60 group (*P* < 0.001). Compared with the Model + Peptide P60 group, cell viability was significantly increased in the Model + YCS + Peptide P60 group (*P* < 0.001) (Figure 7J). These findings indicate that YCS alleviated hyperosmotic co-culture-induced epithelial cell injury, while Peptide P60 further impaired epithelial cell viability. The reduction in cell viability caused by Peptide P60 was partially reversed by combined YCS treatment, suggesting that YCS may partially preserve epithelial cell viability even when Foxp3-associated Treg regulation is pharmacologically disrupted.

#### 3.2.6 YCS reduced ROS accumulation in CD4^+^ T cells under hyperosmotic epithelial–immune co-culture conditions

To evaluate oxidative stress in CD4⁺ T cells under hyperosmotic co-culture conditions, intracellular ROS levels were measured by flow cytometry. Compared with the Control group, the Model group showed a significant increase in ROS levels (*P* < 0.001), indicating enhanced ROS accumulation in CD4⁺ T cells under hyperosmotic co-culture conditions. Compared with the Model group, ROS levels were significantly reduced in the Model + YCS group (*P* = 0.001), and a similar reduction was observed in the Model + AS2863619 group (*P* = 0.002) (Figure 9A).

**Figure 9:**
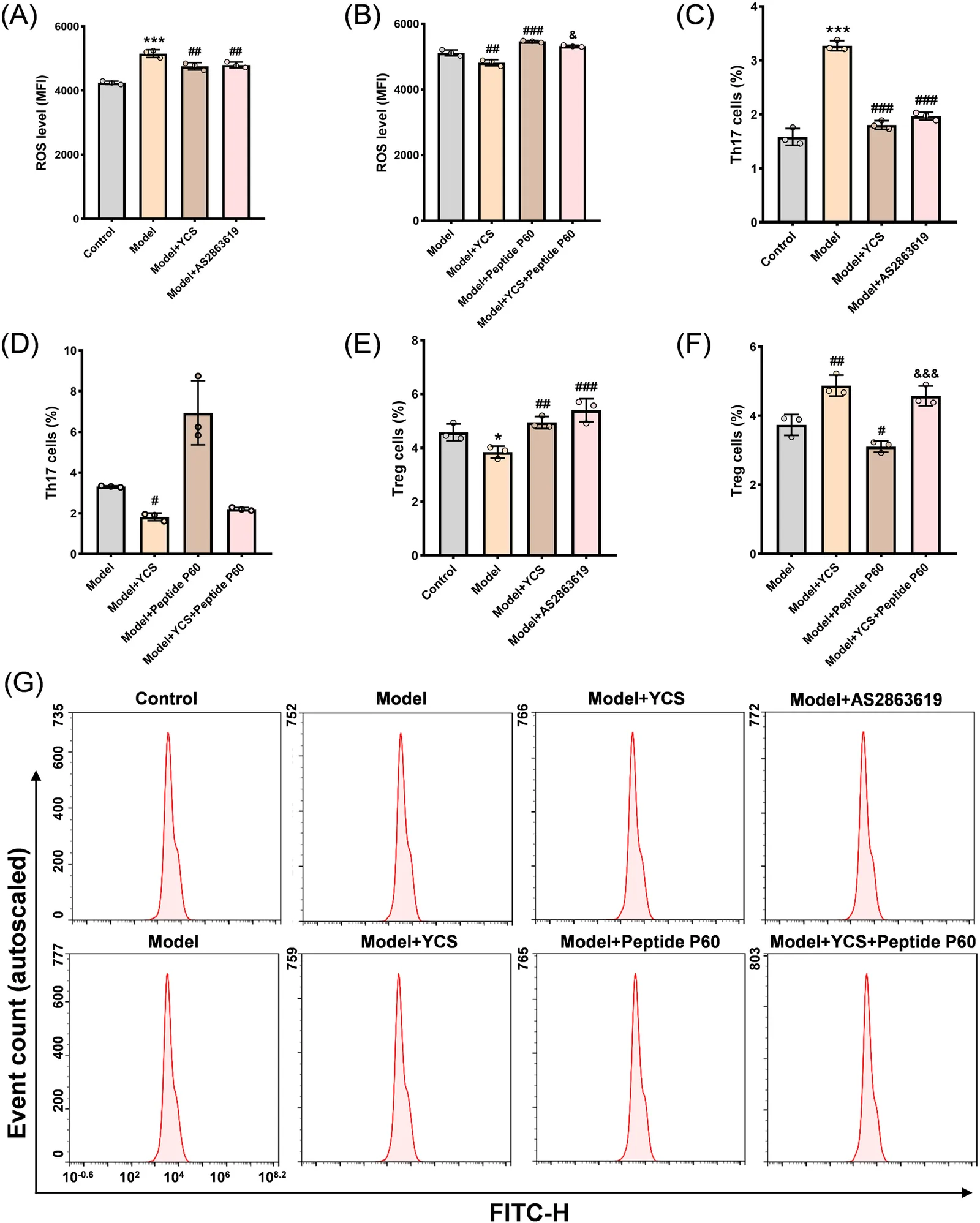
Effects of YCS on ROS accumulation and Th17/Treg differentiation in CD4⁺ T cells under hyperosmotic epithelia l–immune c o-culture conditions. **(A)** Intracellular ROS levels in CD4⁺ T cells after treatment with YCS or AS2863619. **(B)** Intracellular ROS levels in the Peptide P60 intervention experiment. **(C)** Proportion of CD4⁺IL-17A⁺ Th17 cells after treatment with YCS or AS2863619. **(D)** Proportion of CD4⁺IL-17A⁺ Th17 cells in the Peptide P60 intervention experiment. **(E)** Proportion of CD4⁺CD25⁺Foxp3⁺ Treg cells after treatment with YCS or AS2863619. **(F)** Proportion of CD4⁺CD25⁺Foxp3⁺ Treg cells in the Peptide P60 intervention experiment. **(G)** Representative flow cytometry histograms showing DCFH-DA fluorescence intensity for intracellular ROS detection. Data are presented as mean ± SD. For panels **(A–F)**, *n* = 3 independent experiments per group. Representative flow cytometry histograms are shown for panel **(G)**. Statistical significance was determined by one-way ANOVA followed by appropriate *post hoc* tests. *P < 0.05, **P < 0.01, ***P < 0.001 vs. the Control group; ^#^*P* < 0.05, ^##^*P* < 0.01, ^###^*P* < 0.001 vs. the Model group; ^&^*P* < 0.05, ^&&^*P* < 0.01, ^&&&^*P* < 0.001 vs. the Model + Peptide P60 group.

In the Peptide P60 intervention experiment, ROS levels were significantly reduced in the Model + YCS group compared with the Model group (*P* = 0.001), whereas Peptide P60 significantly increased ROS levels (*P* < 0.001). Compared with the Model + Peptide P60 group, ROS levels were significantly decreased in the Model + YCS + Peptide P60 group (*P* = 0.033) (Figure 9B), suggesting that YCS partially attenuated Peptide P60-associated ROS elevation.

Representative flow cytometry histograms supported these quantitative findings. The Model group showed a rightward shift in FITC fluorescence intensity compared with the Control group, whereas YCS or AS2863619 treatment reduced fluorescence intensity. Peptide P60 increased fluorescence intensity, and this change was partially attenuated by combined treatment with YCS (Figure 9G).

These results indicate that YCS reduced oxidative stress in CD4⁺ T cells under hyperosmotic co-culture conditions and attenuated the ROS elevation induced by Peptide P60.

#### 3.2.7 YCS shifted CD4^+^ T-cell differentiation from a Th17-dominant profile toward Treg-associated regulation

To further evaluate the effect of YCS on CD4⁺ T cell differentiation, the proportions of Th17 and Treg cells were analyzed by flow cytometry. Compared with the Control group, the Model group showed a significant increase in the proportion of Th17 cells (*P* < 0.001), indicating that hyperosmotic co-culture conditions promoted CD4⁺ T cell skewing toward the Th17 phenotype. Compared with the Model group, the proportion of Th17 cells was significantly reduced in the Model + YCS group (*P* < 0.001), and a similar decrease was observed in the Model + AS2863619 group (*P* < 0.001) (Figure 9C).

In the Peptide P60 intervention experiment, the proportion of Th17 cells was significantly reduced in the Model + YCS group compared with the Model group (*P* = 0.013). The Model + Peptide P60 group showed an increasing trend in Th17 cells compared with the Model group, but the difference was not statistically significant. Compared with the Model + Peptide P60 group, the Model + YCS + Peptide P60 group showed a decreasing trend in Th17 cells; however, this difference did not reach statistical significance (*P* = 0.103) (Figure 9D).

For Treg cells, the Model group showed a significant decrease compared with the Control group (*P* = 0.019). Compared with the Model group, the proportion of Treg cells was significantly increased in the Model + YCS group (*P* = 0.002), and a similar increase was observed in the Model + AS2863619 group (*P* < 0.001) (Figure 9E). In the Peptide P60 intervention experiment, YCS significantly increased the proportion of Treg cells compared with the Model group (*P* = 0.001), whereas Peptide P60 significantly reduced Treg differentiation (*P* = 0.022). Compared with the Model + Peptide P60 group, the proportion of Treg cells was significantly increased in the Model + YCS + Peptide P60 group (*P* < 0.001) (Figure 9F).

Representative flow cytometry plots further supported these quantitative findings. The proportion of CD4⁺IL-17A⁺ cells was increased in the Model group compared with the Control group, whereas YCS or AS2863619 treatment reduced this proportion. Peptide P60 tended to increase the proportion of CD4⁺IL-17A⁺ cells, while combined treatment with YCS showed a decreasing trend but did not result in a statistically significant reduction compared with Peptide P60 alone (Figure 10A). In parallel, the proportion of CD4⁺CD25⁺Foxp3⁺ cells was reduced in the Model group compared with the Control group, whereas YCS and AS2863619 increased this proportion. Peptide P60 further reduced the proportion of Treg cells, whereas combined YCS treatment increased Treg proportions compared with Peptide P60 alone (Figure 10B).

**Figure 10:**
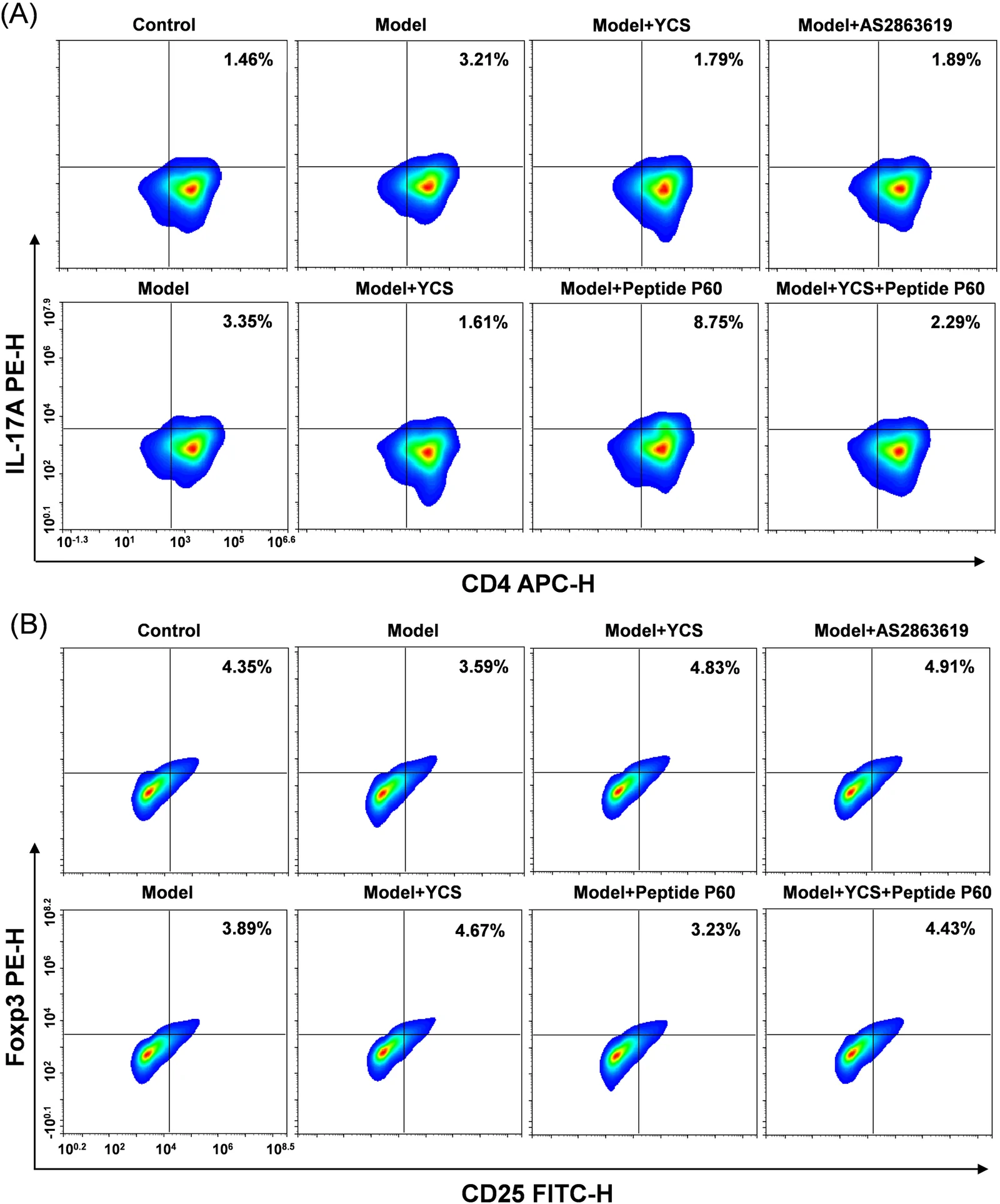
Representative pseudocolor density plots of Th17 and Treg differentiation in CD4⁺ T cells under hyperosmotic epithelial–immune co-culture conditions. **(A)** Representative pseudocolor density plots showing the proportion of CD4⁺IL-17A⁺ Th17 cells. **(B)** Representative pseudocolor density plots showing the proportion of CD4⁺CD25⁺Foxp3⁺ Treg cells. Only the percentage of the upper-right target population is displayed. These plots correspond to the quantitative analyses shown in Figures 9C–F.

Taken together, these results indicate that YCS shifted CD4⁺ T cell differentiation away from a Th17-dominant profile and toward Treg differentiation under hyperosmotic co-culture conditions, thereby improving Th17/Treg imbalance. AS2863619 produced Treg-promoting effects comparable to those of YCS, whereas Peptide P60 mainly impaired Treg-associated immunoregulation.

#### 3.2.8 Immunofluorescence analysis supported the effects of YCS on Foxp3 and IL-17A-associated signals

To further validate the regulatory effects of YCS on Th17/Treg-related phenotypes, immunofluorescence staining was performed to examine Foxp3 and IL-17A signals in CD4⁺ T cells.

Foxp3 staining showed that Foxp3 expression was markedly reduced in the Model group compared with the Control group (*P* < 0.001), indicating suppression of the Treg-related phenotype under hyperosmotic co-culture conditions. Compared with the Model group, Foxp3 expression was increased in the Model + YCS group (*P* < 0.001), with a similar increase observed in the Model + AS2863619 group (*P* < 0.001) (Figure 11A). In the Peptide P60 intervention experiment, Foxp3 expression was increased in the Model + YCS group compared with the Model group (*P* < 0.01), whereas it was decreased in the Model + Peptide P60 group (*P* < 0.001). The Model + YCS + Peptide P60 group showed a higher mean Foxp3 fluorescence intensity than the Model + Peptide P60 group, but this difference did not reach statistical significance (Figure 11B). Representative images further showed weaker Foxp3 fluorescence in the Model group than in the Control group, whereas YCS or AS2863619 treatment enhanced Foxp3 staining. Peptide P60 further reduced Foxp3 fluorescence intensity, whereas combined treatment with YCS increased Foxp3 staining compared with Peptide P60 alone (Figures 11C,D).

**Figure 11:**
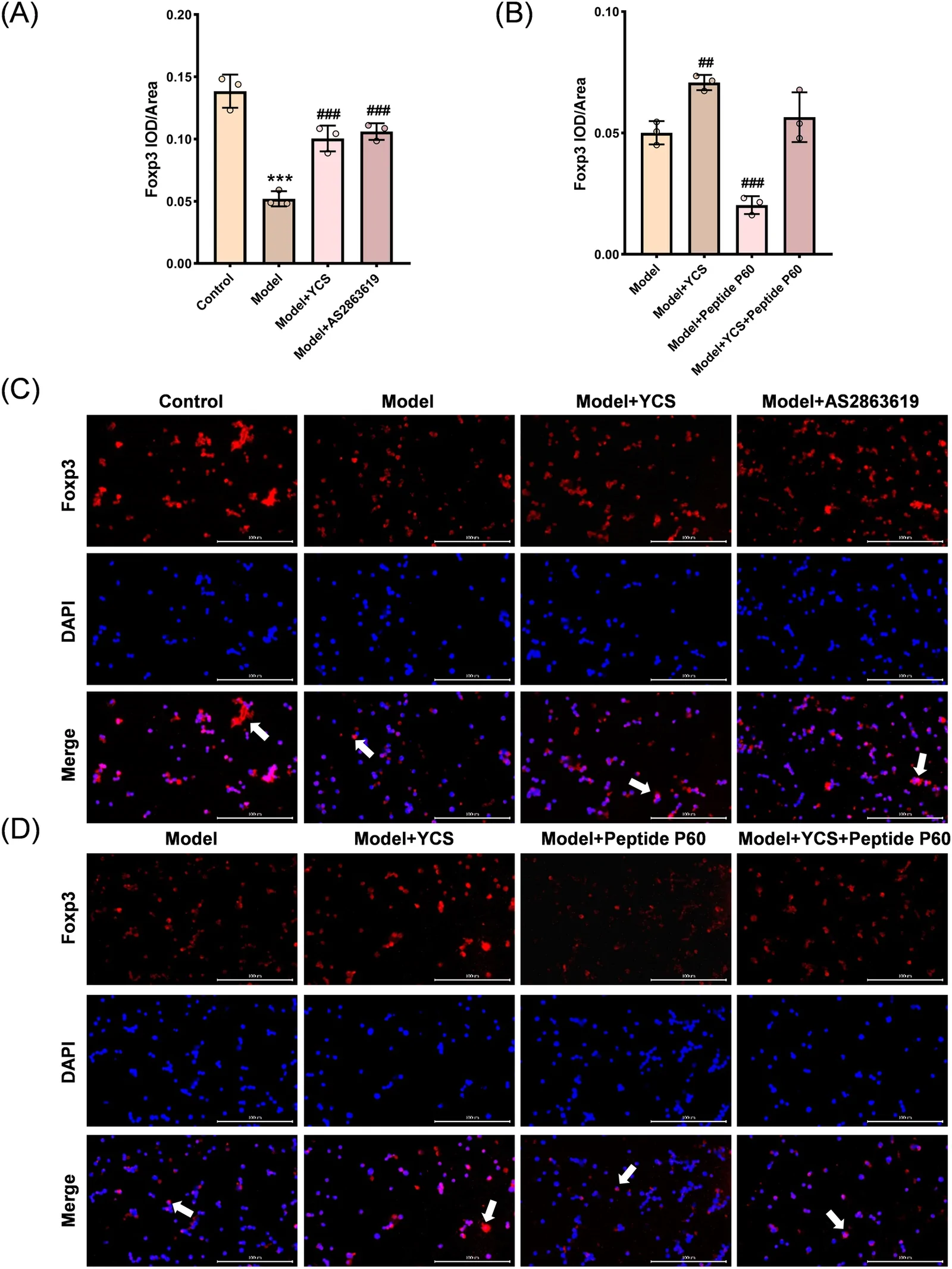
YCS enhanced Foxp3-associated immunofluorescence signals in CD4⁺ T cells under hyperosmotic co-culture conditions. **(A)** Quantitative analysis of Foxp3 fluorescence intensity after treatment with YCS or AS2863619. **(B)** Quantitative analysis of Foxp3 fluorescence intensity in the Peptide P60 intervention experiment. **(C)** Representative immunofluorescence images of Foxp3 staining after treatment with YCS or AS2863619. **(D)** Representative immunofluorescence images of Foxp3 staining in the Peptide P60 intervention experiment. Red fluorescence indicates Foxp3 staining, and blue fluorescence indicates DAPI-stained nuclei. White arrows in the merged images indicate representative Foxp3-associated fluorescence signals overlapping with DAPI-stained nuclei. Scale bar = 100 μm. Data are presented as mean ± SD. For panels A and B, three independent experiments were performed per group. One slide from each independent experiment was analyzed, and three fields were quantified per slide. The mean of the three fields from each slide was used as one independent experimental value for statistical analysis and graphical presentation (*n* = 3). Representative immunofluorescence images are shown for panels C and D. Statistical significance was determined by one-way ANOVA followed by appropriate *post hoc* tests. ***P < 0.001 vs. the Control group; ^##^*P* < 0.01, ^###^*P* < 0.001 vs. the Model group.

IL-17A immunofluorescence staining showed stronger IL-17A-associated signals in the Model group than in the Control group, indicating enhanced IL-17A-related inflammatory activation under hyperosmotic co-culture conditions. Compared with the Model group, IL-17A-associated fluorescence was reduced in the Model + YCS and Model + AS2863619 groups (Figure 12A). In the Peptide P60 intervention experiment, IL-17A-associated fluorescence was significantly reduced in the Model + YCS group compared with the Model group (*P* < 0.01). The Model + Peptide P60 group showed a higher mean fluorescence intensity than the Model group, whereas the Model + YCS + Peptide P60 group showed a lower mean fluorescence intensity than the Model + Peptide P60 group; however, these latter differences did not reach statistical significance (Figure 12B). Representative images showed stronger IL-17A fluorescence in the Model group than in the Control group, whereas YCS and AS2863619 reduced IL-17A staining. Peptide P60 further enhanced IL-17A fluorescence intensity, while combined treatment with YCS attenuated this increase (Figures 12C,D).

**Figure 12:**
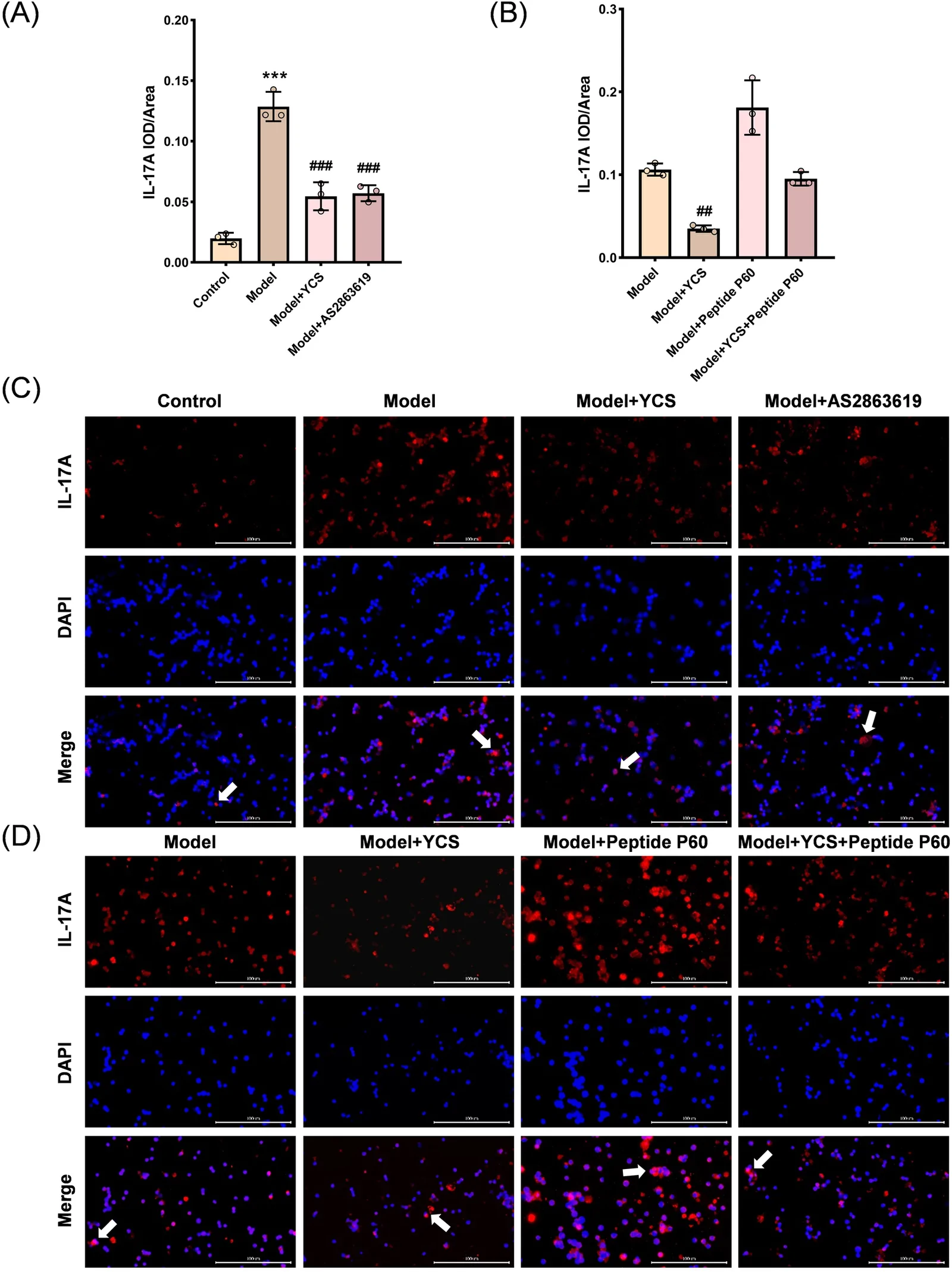
YCS reduced IL-17A-associated immunofluorescence signals in CD4⁺ T cells under hyperosmotic co-culture conditions. **(A)** Quantitative analysis of IL-17A fluorescence intensity after treatment with YCS or AS2863619. **(B)** Quantitative analysis of IL-17A fluorescence intensity in the Peptide P60 intervention experiment. **(C)** Representative immunofluorescence images of IL-17A staining after treatment with YCS or AS2863619. **(D)** Representative immunofluorescence images of IL-17A staining in the Peptide P60 intervention experiment. Red fluorescence indicates IL-17A staining, and blue fluorescence indicates DAPI-stained nuclei. White arrows in the merged images indicate representative IL-17A-associated fluorescence signals in relation to DAPI-stained cells. Scale bar = 100 μm. Data are presented as mean ± SD. For panels A and B, three independent experiments were performed per group. One slide from each independent experiment was analyzed, and three fields were quantified per slide. The mean of the three fields from each slide was used as one independent experimental value for statistical analysis and graphical presentation (*n* = 3). Representative immunofluorescence images are shown for panels C and D. Statistical significance was determined by one-way ANOVA followed by appropriate *post hoc* tests. ***P < 0.001 vs. the Control group; ^##^*P* < 0.01, ^###^*P* < 0.001 vs. the Model group.

Taken together, these immunofluorescence findings indicate that YCS enhanced Foxp3-associated signals and reduced IL-17A-associated fluorescence under hyperosmotic co-culture conditions. Peptide P60 showed an opposite directional pattern, particularly for the Foxp3-associated endpoint, whereas combined YCS treatment partially shifted the mean fluorescence values in the opposite direction, although the corresponding rescue comparisons did not reach statistical significance.

#### 3.2.9 YCS modulated Th17- and Treg-associated transcriptional profiles in CD4^+^ T cells

To further evaluate the regulatory effect of YCS on the Th17/Treg balance at the transcriptional level, RT-qPCR was performed to detect Th17- and Treg-related gene expression in CD4⁺ T cells. For Th17-related genes, RORγt, IL-17A, and IFN-γ mRNA expression was increased in the Model group and reduced after YCS treatment (Figures 13A–C). AS2863619 also reduced RORγt and IFN-γ expression, while IL-17A showed a decreasing trend. These results suggest that YCS suppressed Th17-related transcriptional activation under hyperosmotic co-culture conditions.

**Figure 13:**
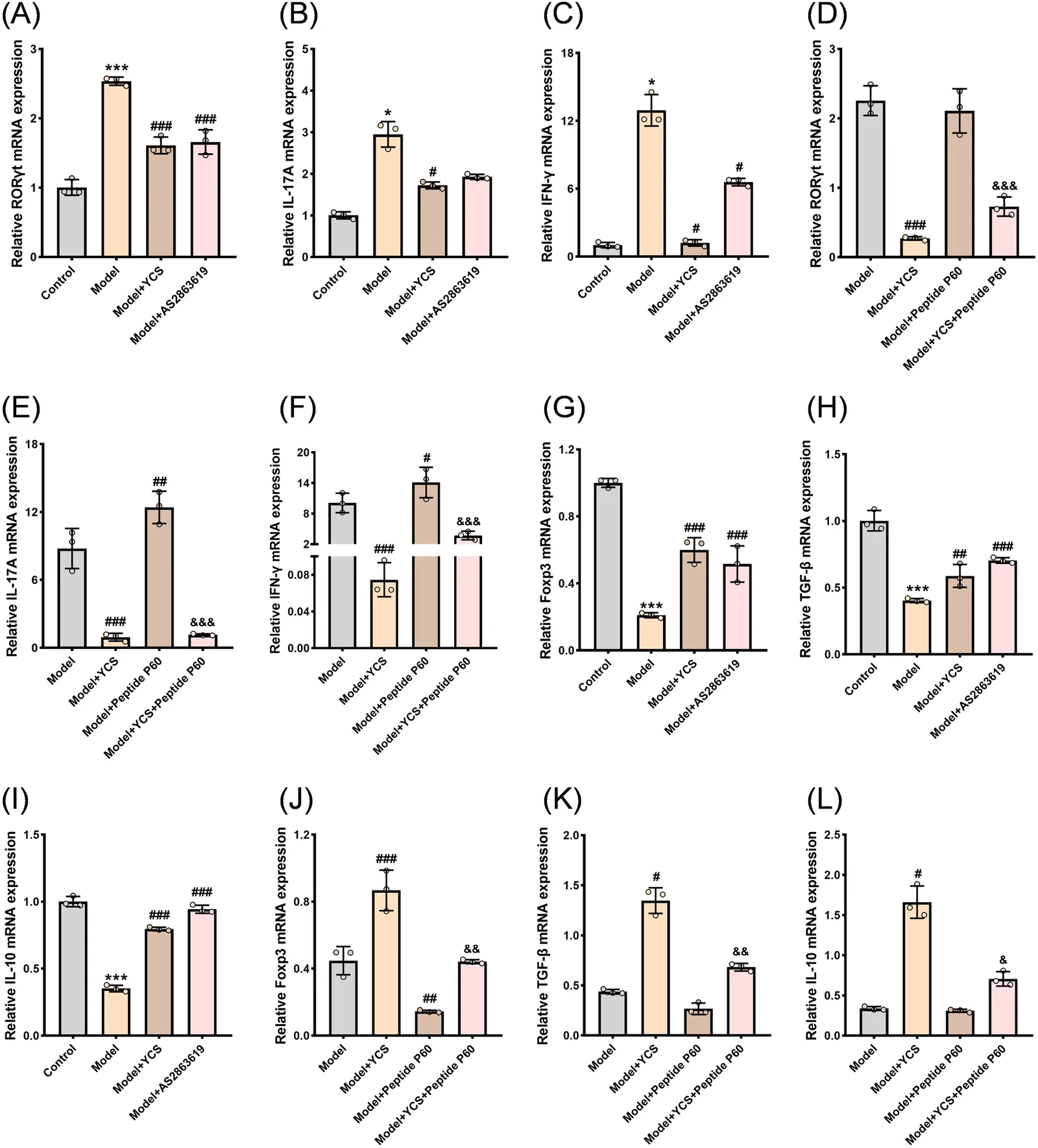
YCS modulated Th17- and Tre g-associated transcriptional profiles in CD4⁺ T cells under hyperosmotic c o-culture conditions. **(A–C)** RT-qPCR analysis of the Th17-related genes RORγt, IL-17A, and IFN-γ after treatment with YCS or AS2863619. **(D–F)** RT-qPCR analysis of RORγt, IL-17A, and IFN-γ in the Peptide P60 intervention experiment. **(G–I)** RT-qPCR analysis of the Treg-related genes Foxp3, TGF-β, and IL-10 after treatment with YCS or AS2863619. **(J–L)** RT-qPCR analysis of Foxp3, TGF-β, and IL-10 in the Peptide P60 intervention experiment. Data are presented as mean ± SD. For panels **(A–L)**, *n* = 3 independent experiments per group. Statistical significance was determined by one-way ANOVA followed by appropriate *post hoc* tests. *P < 0.05, ***P < 0.001 vs. the Control group; ^#^*P* < 0.05, ^##^*P* < 0.01, ^###^*P* < 0.001 vs. the Model group; ^&^*P* < 0.05, ^&&^*P* < 0.01, ^&&&^*P* < 0.001 vs. the Model + Peptide P60 group.

In the Peptide P60 intervention experiment, YCS significantly decreased RORγt, IL-17A, and IFN-γ expression compared with the Model group (all *P* < 0.001). Peptide P60 significantly increased IL-17A and IFN-γ expression (both *P* < 0.05), whereas RORγt expression did not differ significantly between the Model + Peptide P60 group and the Model group. Combined treatment with YCS significantly reduced RORγt, IL-17A, and IFN-γ expression compared with the Model + Peptide P60 group (all *P* < 0.001) (Figures 13D–F). These results indicate that YCS suppressed Th17-related pro-inflammatory gene expression and attenuated Peptide P60-induced transcriptional changes.

For Treg-related genes, Foxp3, TGF-β, and IL-10 expression was reduced in the Model group and increased after YCS treatment. AS2863619 produced a similar upregulatory effect (Figures 13G–I).

In the Peptide P60 intervention experiment, YCS significantly increased Foxp3, TGF-β, and IL-10 expression compared with the Model group (all *P* < 0.05). Peptide P60 significantly reduced Foxp3 expression (*P* = 0.001), while TGF-β and IL-10 showed non-significant decreasing trends. Combined treatment with YCS significantly increased Foxp3, TGF-β, and IL-10 expression compared with the Model + Peptide P60 group (all *P* < 0.05) (Figures 13J–L). Together, these findings suggest that YCS shifted CD4⁺ T cells away from a Th17-related transcriptional profile and toward a Treg-associated regulatory profile under hyperosmotic co-culture conditions.

#### 3.2.10 YCS attenuated inflammatory transcriptional changes in lacrimal gland epithelial cells

To further evaluate the effect of YCS on inflammation-related gene expression in lacrimal gland epithelial cells under hyperosmotic co-culture conditions, RT-qPCR was performed to detect IL-17A, TGF-β, and IL-10 mRNA expression in epithelial cells from the upper chamber.

Compared with the Control group, the Model group showed significantly increased IL-17A expression (*P* = 0.017) and decreased TGF-β and IL-10 expression (both *P* < 0.001), indicating that hyperosmotic co-culture conditions shifted lacrimal gland epithelial cells toward a more inflammatory transcriptional profile. Compared with the Model group, YCS significantly reduced IL-17A expression (*P* = 0.049) and increased TGF-β and IL-10 expression (both *P* < 0.01). AS2863619 significantly increased TGF-β and IL-10 expression (both *P* < 0.001), whereas IL-17A expression showed a decreasing trend without statistical significance (Figures 14A–C).

**Figure 14:**
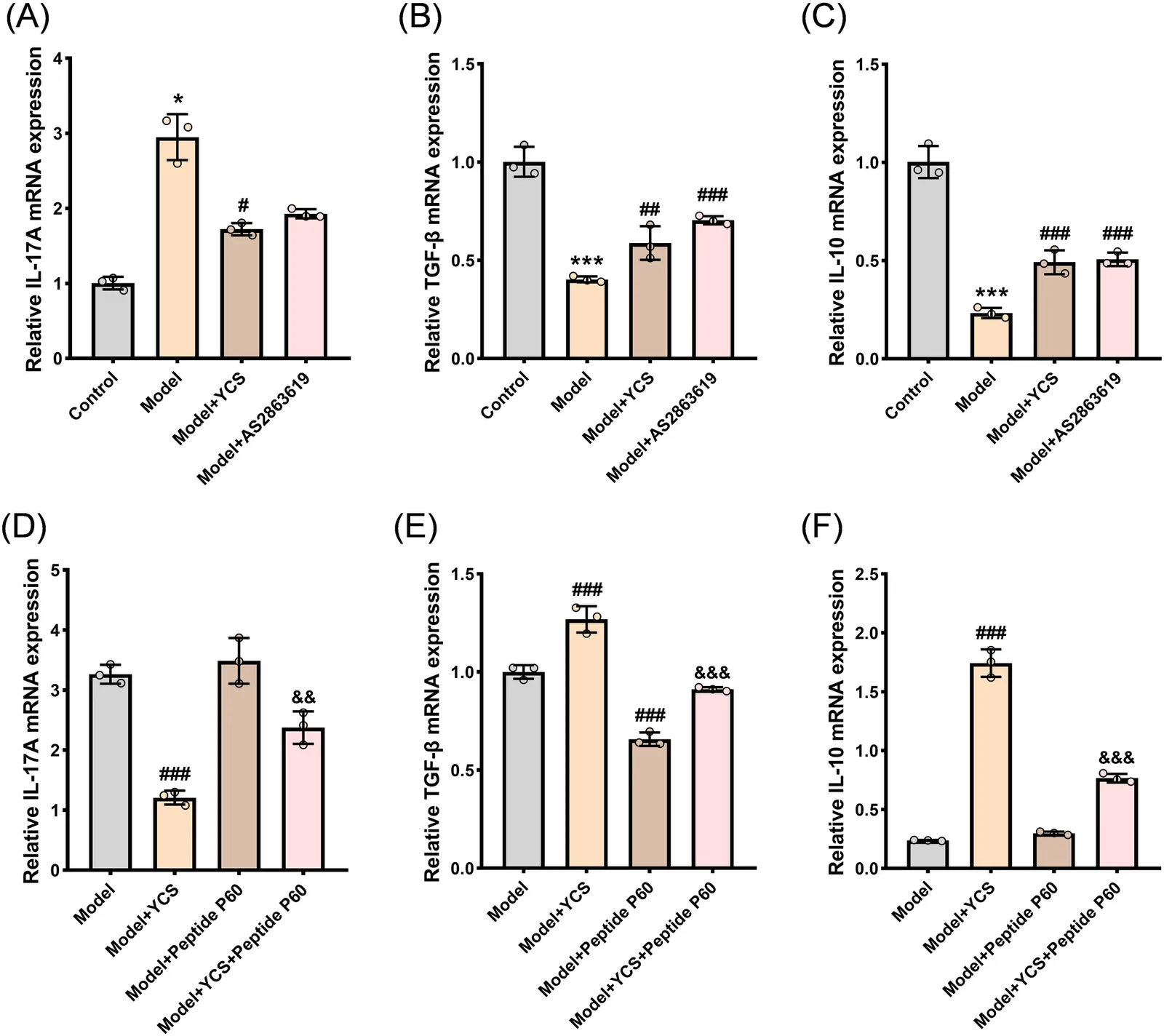
YCS attenuated inflammatory transcriptional changes in lacrimal gland epithelial cells under hyperosmotic co-culture conditions. **(A–C)** RT-qPCR analysis of IL-17A, TGF-β, and IL-10 mRNA expression after treatment with YCS or AS2863619. **(D–F)** RT-qPCR analysis of IL-17A, TGF-β, and IL-10 mRNA expression in the Peptide P60 intervention experiment. Data are presented as mean ± SD. For panels **(A–F)**, *n* = 3 independent experiments per group. Statistical significance was determined by one-way ANOVA followed by appropriate *post hoc* tests. For panels A–C, *P < 0.05, ***P < 0.001 vs. the Control group; for all panels, ^#^*P* < 0.05, ^##^*P* < 0.01, ^###^*P* < 0.001 vs. the Model group; ^&&^*P* < 0.01, ^&&&^*P* < 0.001 vs. the Model + Peptide P60 group.

In the Peptide P60 intervention experiment, YCS significantly reduced IL-17A expression and increased TGF-β and IL-10 expression compared with the Model group (all *P* < 0.001). Peptide P60 significantly reduced TGF-β expression (*P* < 0.001), whereas IL-17A and IL-10 showed no significant changes. Compared with the Model + Peptide P60 group, combined treatment with YCS significantly decreased IL-17A expression (*P* = 0.001) and increased TGF-β and IL-10 expression (both *P* < 0.001) (Figures 14D–F).

These findings suggest that YCS attenuated inflammatory transcriptional changes in lacrimal gland epithelial cells under hyperosmotic co-culture conditions.

## 4 Discussion

DED is increasingly recognized as an immune-mediated ocular surface disorder in which tear film instability, epithelial injury, and immune dysregulation reinforce one another (15, 16). In the present study, YGJ improved tear secretion and tear film stability, reduced corneal fluorescein staining, and alleviated corneal and lacrimal gland histopathological injury, while meibomian gland and conjunctival tissues showed partial morphological preservation in BAC-induced dry eye rats. These *in vivo* findings suggest that YGJ improved ocular surface signs and mitigated lacrimal gland inflammatory injury, with partial morphological preservation in the meibomian gland and conjunctiva. Importantly, YGJ treatment was associated with lower lacrimal gland Th17 proportions, higher Treg proportions, coordinated changes in Th17/Treg-related molecular markers, and reduced STAT3 phosphorylation. Together, these findings provide preliminary evidence of an association between YGJ treatment, improved lacrimal gland immune homeostasis, and improvement of ocular surface signs (17, 18). Figure 15 summarizes the parallel treatment-associated findings from the in vivo and in vitro experiments.

**Figure 15:**
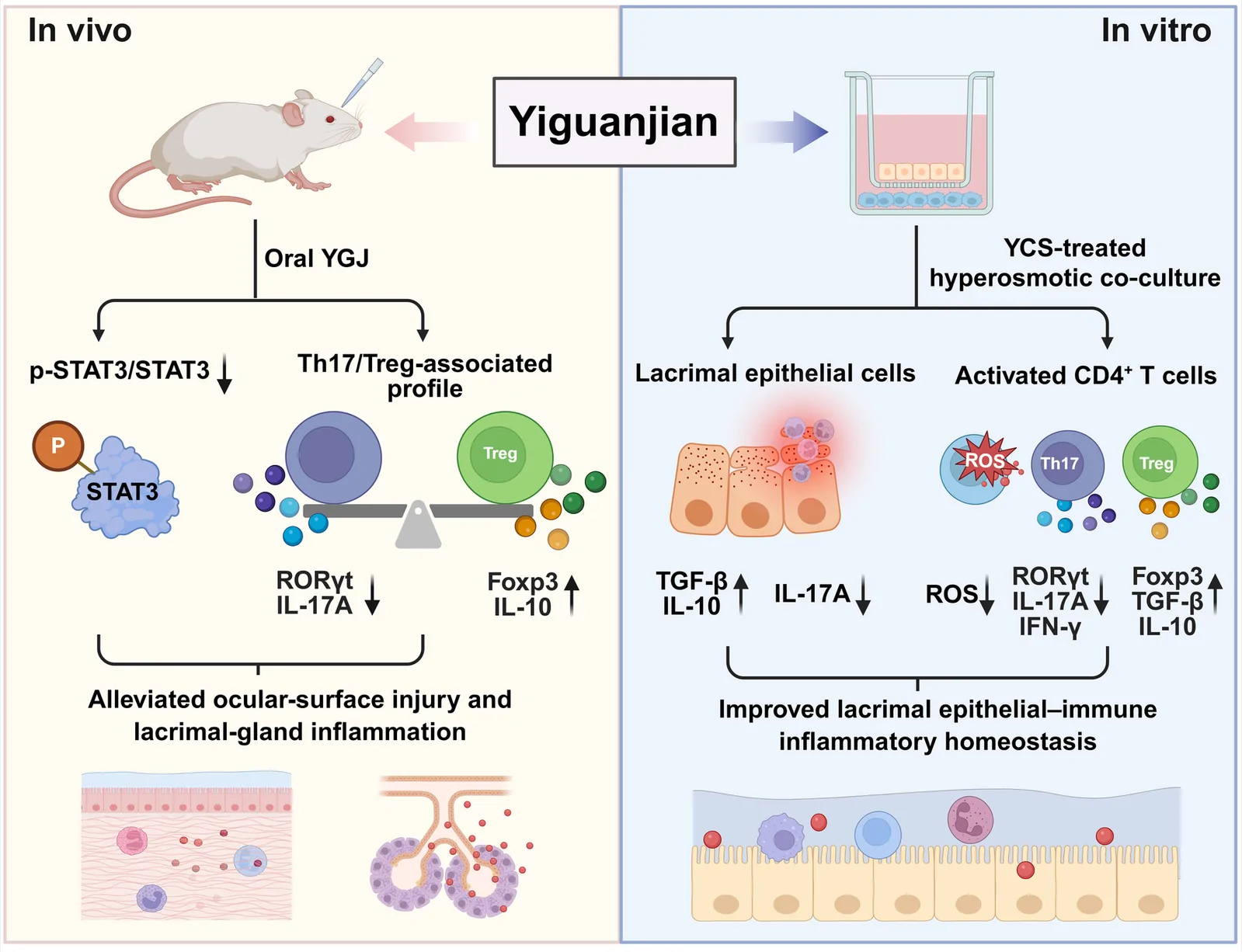
Schematic summary of the proposed biological framework for YGJ/YC S-associated effects in experimental dry eye disease. *In vivo*, oral Yiguanjian (YGJ) treatment improved ocular-surface signs and alleviated lacrimal-gland inflammatory injury in benzalkonium chloride (BAC)-induced dry eye rats. These changes were accompanied by a reduced lacrimal-gland p-STAT3/STAT3 ratio, decreased Th17 proportions and Th17-associated markers, and increased Treg proportions and Treg-associated markers, consistent with partial improvement of Th17/Treg-related immune homeostasis. *In vitro*, Yiguanjian-containing serum (YCS) improved lacrimal-gland epithelial cell viability and attenuated inflammatory transcriptional changes in epithelial cells under hyperosmotic co-culture conditions. In CD4⁺ T cells, YCS reduced ROS accumulation and Th17-associated responses while enhancing Foxp3/Treg-associated regulation. The schematic integrates parallel treatment-associated findings from the two experimental systems; because STAT3 phosphorylation was evaluated *in vivo* and ROS in CD4⁺ T cells *in vitro*, no causal sequence among STAT3 activation, oxidative stress, and Th17/Treg regulation is inferred. BAC, benzalkonium chloride; YGJ, Yiguanjian; YCS, Yiguanjian-containing serum; p-STAT3, phosphorylated signal transducer and activator of transcription 3; Th17, T helper 17; Treg, regulatory T; ROS, reactive oxygen species; RORγt, retinoic acid receptor-related orphan receptor-γt; IL-17A, interleukin-17A; Foxp3, forkhead box P3; IL-10, interleukin-10; TGF-β, transforming growth factor-β; IFN-γ, interferon-γ. Created in BioRender. zhong, Y. (2026) https://BioRender.com/yfz15jy.

The less prominent morphological changes in the meibomian gland and conjunctiva may reflect differences in tissue function, injury pattern, and repair response. Interpretation is also influenced by the depth of tissue assessment: the lacrimal gland was examined by histology, flow cytometry, RT-qPCR, and western blotting, whereas the meibomian gland and conjunctiva were evaluated mainly by histology. The apparent tissue differences should therefore be interpreted cautiously. Differences in local tissue exposure or cellular responsiveness may also contribute, although tissue-specific pharmacokinetics and comparative cellular sensitivity were not assessed in this study. Further tissue-distribution and parallel tissue-specific functional studies are needed to clarify these differences.

As a key component of the lacrimal functional unit, the lacrimal gland contributes to aqueous tear secretion and participates in immune regulation relevant to DED pathogenesis (19, 20). Dysfunction of the lacrimal gland can impair tear secretion and alter the inflammatory milieu, thereby sustaining ocular surface damage (21, 22). In our BAC-induced model, lacrimal gland tissues showed structural disruption, interstitial expansion, and increased accumulation of densely stained nuclei suggestive of inflammatory cell infiltration, accompanied by Th17/Treg imbalance. These changes were attenuated after YGJ treatment, especially in the medium- and high-dose groups. This pattern supports the view that lacrimal gland immune dysregulation contributes to DED progression and that restoration of local immune homeostasis may be an important component of YGJ-mediated protection (23, 24).

Th17/Treg imbalance is recognized as an important immunological feature of chronic ocular surface inflammation in DED (25, 26). Th17 cells promote tissue inflammation mainly through IL-17A-associated effector responses, whereas Treg cells restrain excessive immune activation and maintain immune tolerance (27). In this study, YGJ decreased Th17 proportions and increased Treg proportions in lacrimal gland tissues. Consistently, YGJ downregulated RORγt and IL-17A while upregulating Foxp3 and IL-10. These findings suggest that YGJ shifted the lacrimal gland immune profile away from a Th17-dominant inflammatory state and toward Treg-associated immune regulation. The concomitant reduction in the p-STAT3/STAT3 ratio further supports an association between improved Th17/Treg-related immune homeostasis and reduced STAT3 activation (28). However, because STAT3 was not specifically manipulated *in vivo*, these parallel changes do not establish that reduced STAT3 phosphorylation caused the restoration of the Th17/Treg balance.

Because traditional herbal formulae undergo absorption and metabolic transformation after oral administration, serum pharmacochemistry can help relate oral administration to circulating chemical exposure (29). The annotation results showed that YCS contained a chemically diverse profile, with shikimates and phenylpropanoids representing the largest annotated group. Several representative candidate annotations correspond to compounds with previously reported pharmacological activities relevant to the present findings. Decursinol angelate has been shown to suppress Th17 differentiation, RORγt expression, and IL-17 production and to attenuate Th17-associated inflammation in experimental colitis (30). Fraxin reduced pro-inflammatory cytokine production and inhibited NF-κB/NLRP3-related signaling in an LPS-induced inflammatory model (31), whereas scutellarin showed anti-inflammatory and antioxidant effects associated with regulation of Nrf2/HO-1 and AKT/p38 MAPK/NF-κB signaling (32).

These reports provide biological plausibility for several candidates in the YCS profile but do not establish their pharmacodynamic contribution in the present study. The present untargeted analysis also does not quantify their circulating exposure. Neither the numerical predominance of a structural class nor the observed serum detection patterns can determine which individual constituent or chemical class is primarily responsible for the effects of YCS. In addition, the precise origins of the YCS-only features cannot be resolved by the present untargeted analysis. Pharmacokinetic profiling and computational prioritization may help narrow the candidate pool (33–35), but targeted quantification, exposure–effect analysis, and functional validation of representative compounds, individually and in defined combinations, will still be required to identify the principal active constituents.

To further examine how serum-associated factors following YGJ administration may modulate epithelial–immune interactions, we established a hyperosmotic non-contact co-culture system comprising rat lacrimal gland epithelial cells and activated CD4⁺ T cells. This design was based on evidence that lacrimal gland epithelial cells can actively shape immune responses and that CD4⁺ T cell-mediated adaptive immunity contributes to DED-related inflammation (36, 37). Although this model cannot fully reproduce the complexity of DED *in vivo*, it provides a controlled system for evaluating soluble mediator-dependent communication between lacrimal gland epithelial cells and CD4⁺ T cells under hyperosmotic stress (38). Under hyperosmotic co-culture conditions, epithelial cell viability decreased, ROS accumulated in CD4⁺ T cells, Th17-related responses were enhanced, and Treg-associated regulation was weakened, consistent with evidence that hyperosmolar stress can induce epithelial inflammatory activation, oxidative stress, and immune-cell-driven inflammatory amplification in DED (39, 40). YCS reversed these changes to varying degrees, suggesting that serum-associated factors following YGJ administration may regulate both epithelial injury and immune-cell polarization within a lacrimal gland epithelial cell–CD4⁺ T cell inflammatory microenvironment.

Foxp3 is essential for Treg lineage stability and suppressive function, and disruption of Foxp3-associated Treg regulation can weaken immune tolerance and favor inflammatory persistence (41, 42). In the present study, YCS increased Foxp3 expression, enhanced Treg differentiation, and suppressed IL-17A-associated inflammatory signals in CD4⁺ T cells. AS2863619, a CDK8/19 inhibitor reported to induce Foxp3-expressing Treg cells, produced effects comparable to those of YCS, including enhanced Treg-related responses, reduced ROS accumulation, and improved epithelial cell viability (43). In contrast, Peptide P60, a Foxp3 inhibitory peptide reported to impair Treg activity, reduced Foxp3-associated immunoregulation, promoted ROS accumulation, and aggravated epithelial injury-related changes (44, 45). Combined YCS treatment attenuated several Peptide P60-induced effects, supporting the involvement of Foxp3-associated regulation in the action of YCS, while not excluding additional Foxp3-independent mechanisms.

It should be noted that Foxp3-associated regulation may not fully explain all effects of YCS. In the Peptide P60 intervention experiment, YCS significantly restored Treg-related changes, whereas its effect on Th17 proportions did not always reach statistical significance. This observation suggests that YCS may influence the Th17/Treg axis through Foxp3-associated mechanisms together with additional Foxp3-independent pathways (46, 47). Oxidative stress may represent one such mechanism. Excessive ROS can amplify inflammatory signaling and influence T cell polarization, whereas ROS reduction may help restore immune balance (48, 49). The finding that YCS reduced ROS accumulation in CD4⁺ T cells, even during Peptide P60-mediated disruption of Foxp3-associated regulation, suggests that antioxidant effects may cooperate with Treg-associated immune modulation to reshape the inflammatory response (50).

In addition to immune-cell changes, lacrimal gland epithelial cells also responded to hyperosmotic inflammatory stress. In the co-culture model, hyperosmotic stimulation reduced epithelial cell viability and shifted epithelial gene expression toward a more inflammatory profile, characterized by increased IL-17A and reduced TGF-β and IL-10 expression. This is consistent with evidence that hyperosmolarity can drive epithelial inflammatory injury in DED (51). YCS improved epithelial cell viability and partially normalized these transcriptional changes. These findings suggest that epithelial cells are not merely passive targets of inflammatory injury but may actively participate in local inflammatory circuits, consistent with single-cell evidence showing an epithelial cell-centered pro-inflammatory microenvironment involving CD4^+^ T cell interactions in DED (52). By modulating both epithelial responses and CD4⁺ T cell immune polarization, YCS may interrupt the reciprocal amplification between epithelial injury and immune inflammation, a process in which IL-17A-associated immune responses have been implicated (53).

Importantly, the STAT3 and ROS findings were obtained from different experimental systems. STAT3 phosphorylation was assessed in lacrimal gland tissues from YGJ-treated rats, whereas intracellular ROS was measured in CD4⁺ T cells in the YCS-treated hyperosmotic co-culture model. STAT3 phosphorylation was not assessed in the co-culture model, and ROS was not measured in the same *in vivo* lacrimal gland samples. Their causal relationship therefore remains unresolved, and the current data do not establish direct regulation of STAT3 by YCS or mediation of one endpoint by the other. Whether STAT3 signaling is required for the protective effects of YCS in the co-culture model also remains unresolved.

Several limitations should be acknowledged. First, the terminal lacrimal gland flow cytometry, RT-qPCR, and western blot analyses were based on three independent animals per group, and the primary-cell assays were based on three independent experiments. These sample sizes limit statistical precision and the assessment of inter-animal and inter-experiment variability; the corresponding findings should therefore be considered preliminary and confirmed in larger independent animal cohorts and additional independent primary-cell experiments. Second, the present study did not include validation in human samples, and the *in vivo* findings were obtained only in a BAC-induced DED model. Although the BAC model reproduces ocular surface injury and inflammation, it does not capture the etiological heterogeneity of human DED. In addition, the *in vitro* co-culture system cannot recapitulate the full complexity of DED *in vivo*, including tear film dynamics, lacrimal functional-unit regulation, innervation, vascular factors, and resident immune-cell networks (54, 55). Validation in human samples and additional DED models is therefore needed. Third, although UHPLC-Q-Orbitrap HRMS characterized candidate serum pharmacochemistry annotations in YCS, the origins of some features and the relative contributions of individual compounds and chemical classes were not determined. No individual candidate compound was directly tested for its contribution to the observed effects. Targeted quantification, activity-guided fractionation, and functional validation of representative compounds alone or in defined combinations will therefore be required to identify the principal active constituents (56, 57). Fourth, the causal mechanisms underlying the immunomodulatory effects of YGJ/YCS remain incompletely resolved. AS2863619 and Peptide P60 provided pharmacological evidence supporting the involvement of Foxp3-associated regulation; however, these interventions do not establish Foxp3 as the sole or direct mechanism underlying the effects of YCS, especially given the plasticity and context-dependent regulation of Treg biology (58). In addition, no STAT3-specific perturbation or rescue experiment was performed to determine whether reduced STAT3 phosphorylation contributes causally to restoration of the Th17/Treg balance. The STAT3 and ROS endpoints were also assessed in different experimental systems. Therefore, the causal relationships among STAT3 activation, Th17/Treg regulation, and oxidative stress remain unresolved, and whether STAT3 signaling is required for the protective effects of YGJ/YCS remains to be established. Future studies should integrate chemical-component validation, matched assessment of STAT3 activation and ROS within the same experimental system, and validated cell-specific STAT3 gain-of-function, loss-of-function, and rescue approaches with confirmation of target engagement, together with cytokine secretion analysis, to clarify the mechanisms by which serum-associated constituents following YGJ administration regulate lacrimal gland immune homeostasis in DED.

In conclusion, this study shows that YGJ attenuated experimental DED-related inflammatory injury *in vivo* and that YCS modulated hyperosmotic inflammatory responses in a lacrimal gland epithelial cell–CD4⁺ T-cell co-culture system. *In vivo*, YGJ treatment was associated with reduced lacrimal gland STAT3 phosphorylation and changes consistent with partial improvement of Th17/Treg-related immune homeostasis. *In vitro*, YCS improved epithelial cell viability, reduced ROS accumulation in CD4⁺ T cells, suppressed Th17-associated inflammatory responses, and enhanced Foxp3-associated Treg immunoregulation. The present data do not establish a causal sequence between the STAT3 and ROS findings or demonstrate that STAT3 signaling is a necessary mediator of the protective effects of YCS. These findings support further investigation of YGJ/YCS as potential modulators of ocular surface inflammation, lacrimal gland immune dysregulation, and epithelial–immune inflammatory crosstalk.

## Data Availability

The original contributions presented in the study are included in the article/Supplementary Material, further inquiries can be directed to the corresponding author.
