# Supplementary material for: Yiguanjian alleviates ocular surface inflammation and lacrimal gland immune dysregulation in experimental dry eye disease: evidence from in vivo and in vitro models
Source: Front Med (Lausanne). 2026 Sep 28;13:1915720. doi: 10.3389/fmed.2026.1915720 (PMC13654703; doi:10.3389/fmed.2026.1915720)
Supplement: Supplementary file 1 [file Image1.pdf]

Supplementary material captions

Supplementary Figure S1.

Representative replicate total ion chromatograms of Yiguanjian decoction (YGJ) and Yiguanjian-containing serum (YCS) acquired by UHPLC-Q-Orbitrap HRMS in negative and positive ion modes. The merged PDF contains three replicate chromatograms for each sample type and ion mode, including NEG YCS1–3, NEG YGJ1–3, POS YCS1–3, and POS YGJ1–3. These chromatograms demonstrate consistent chromatographic profiles across replicates and support the analytical stability and reproducibility of the LC–MS/MS analysis.

Supplementary Table S1.

Primer sequences used for RT-qPCR analysis of rat lacrimal gland tissues in the in vivo experiment.

Supplementary Table S2.

Primer sequences used for RT-qPCR analysis of $CD4^+$ T cells and lacrimal gland epithelial cells in the in vitro experiment.

Supplementary Table S3.

Candidate annotations assigned to LC–MS features detected across Yiguanjian decoction, blank serum, and Yiguanjian-containing serum by UHPLC-Q-Orbitrap HRMS.

Supplementary Table S4.

Candidate serum pharmacochemistry annotations corresponding to LC–MS features detected in Yiguanjian-containing serum by UHPLC-Q-Orbitrap HRMS. The table includes chemical superclass, class, subclass, serum pharmacochemistry category, LC–MS feature ID, MS/MS composite matching score, and annotation notes for 67 candidate annotations corresponding to 56 distinct LC–MS features.

Supplementary Table S5.

Assignments of numbered peaks in the representative positive- and negative-ion total ion chromatograms shown in Figures 5 and 6. Peak assignments were based on MS/MS spectral matching and database annotation and represent candidate chromatographic peak annotations rather than independently confirmed blood-absorbed components.

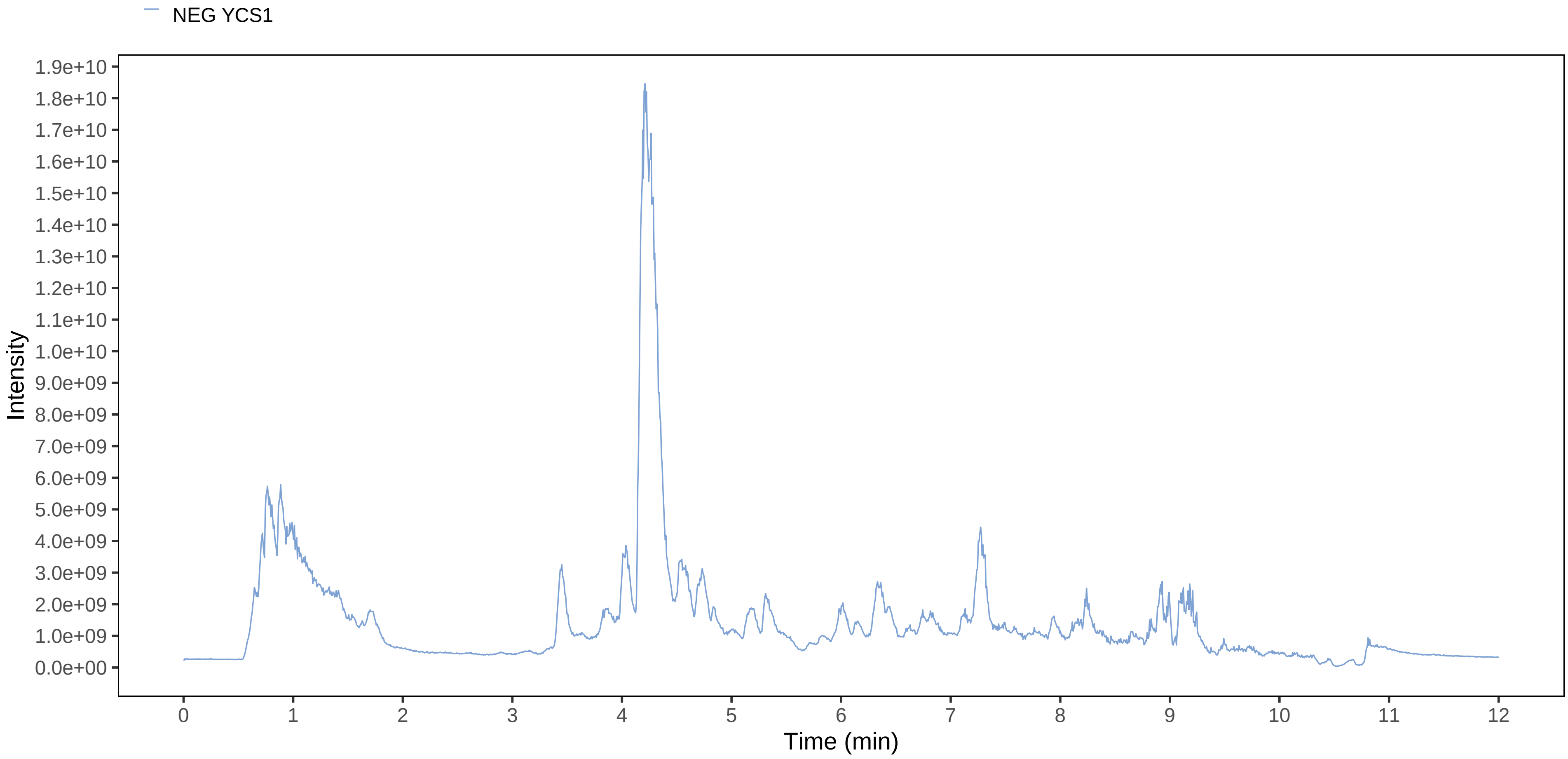
NEG YCS1
Intensity
Time (min)
1.9e+10
1.8e+10
1.7e+10
1.6e+10
1.5e+10
1.4e+10
1.3e+10
1.2e+10
1.1e+10
1.0e+10
9.0e+09
8.0e+09
7.0e+09
6.0e+09
5.0e+09
4.0e+09
3.0e+09
2.0e+09
1.0e+09
0.0e+00
0
1
2
3
4
5
6
7
8
9
10
11
12

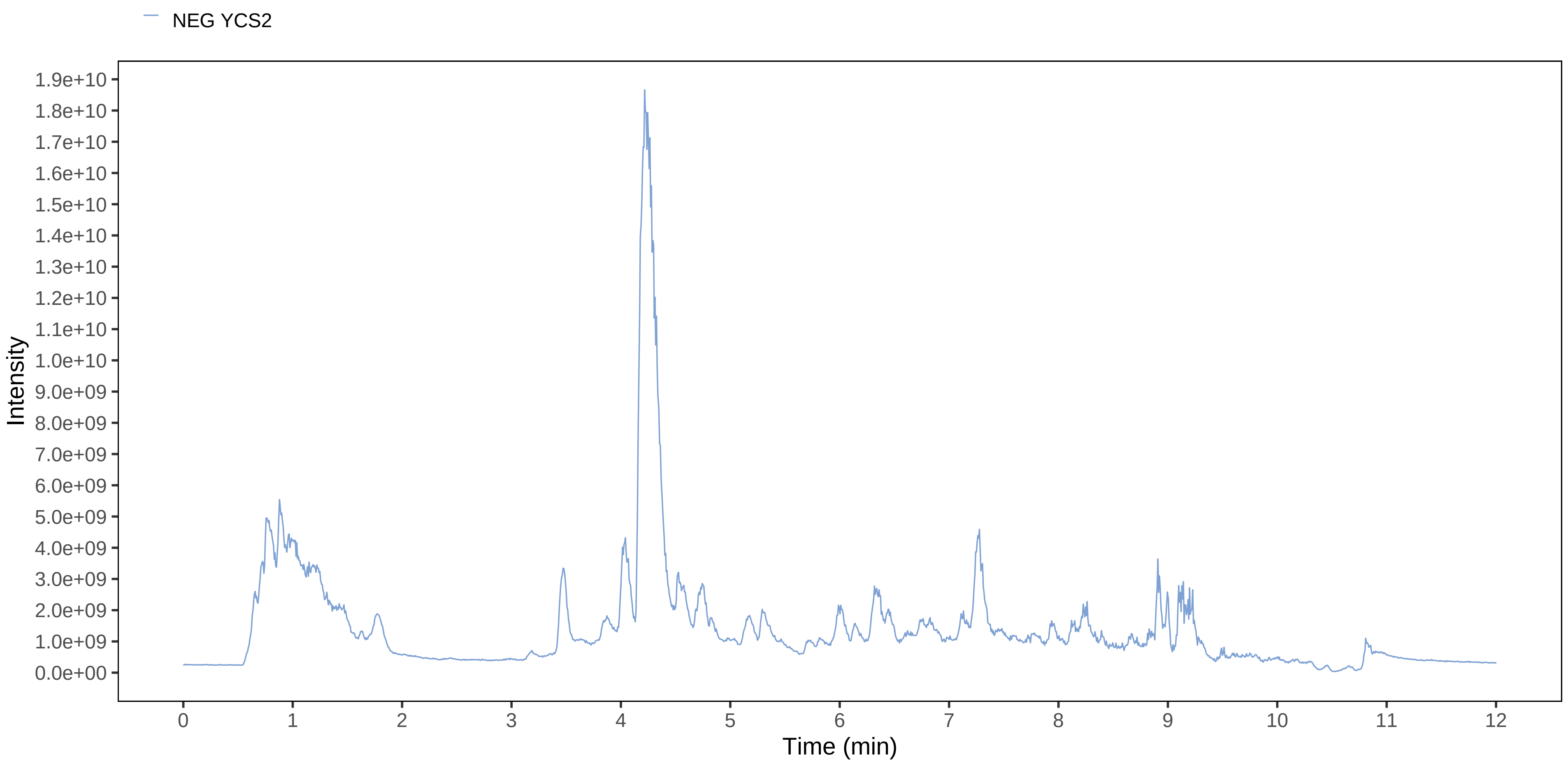
NEG YCS2
Intensity
1.9e+10
1.8e+10
1.7e+10
1.6e+10
1.5e+10
1.4e+10
1.3e+10
1.2e+10
1.1e+10
1.0e+10
9.0e+09
8.0e+09
7.0e+09
6.0e+09
5.0e+09
4.0e+09
3.0e+09
2.0e+09
1.0e+09
0.0e+00
0
1
2
3
4
5
6
7
8
9
10
11
12
Time (min)

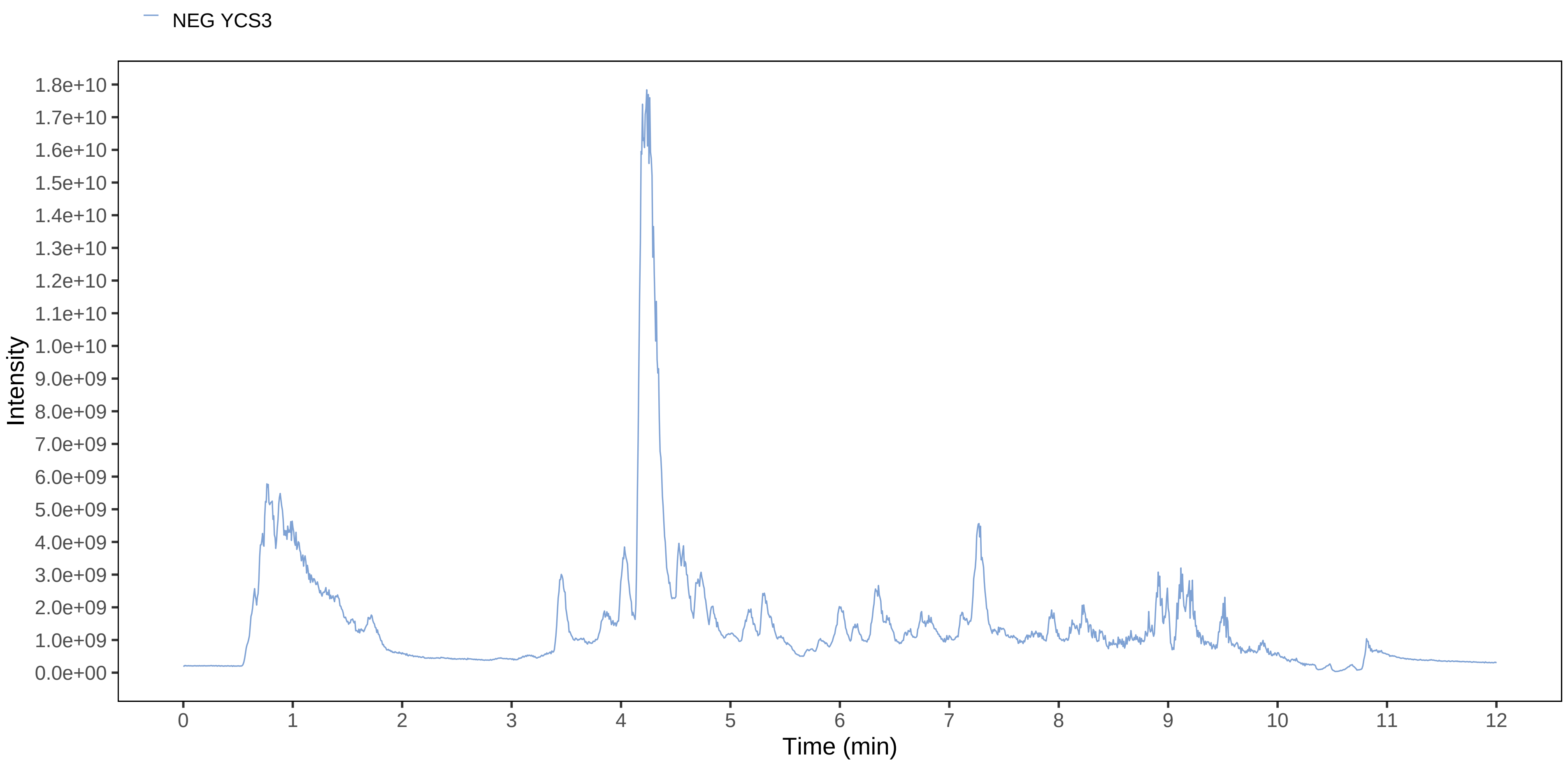
NEG YCS3
Intensity
1.8e+10
1.7e+10
1.6e+10
1.5e+10
1.4e+10
1.3e+10
1.2e+10
1.1e+10
1.0e+10
9.0e+09
8.0e+09
7.0e+09
6.0e+09
5.0e+09
4.0e+09
3.0e+09
2.0e+09
1.0e+09
0.0e+00
0
1
2
3
4
5
6
7
8
9
10
11
12
Time (min)

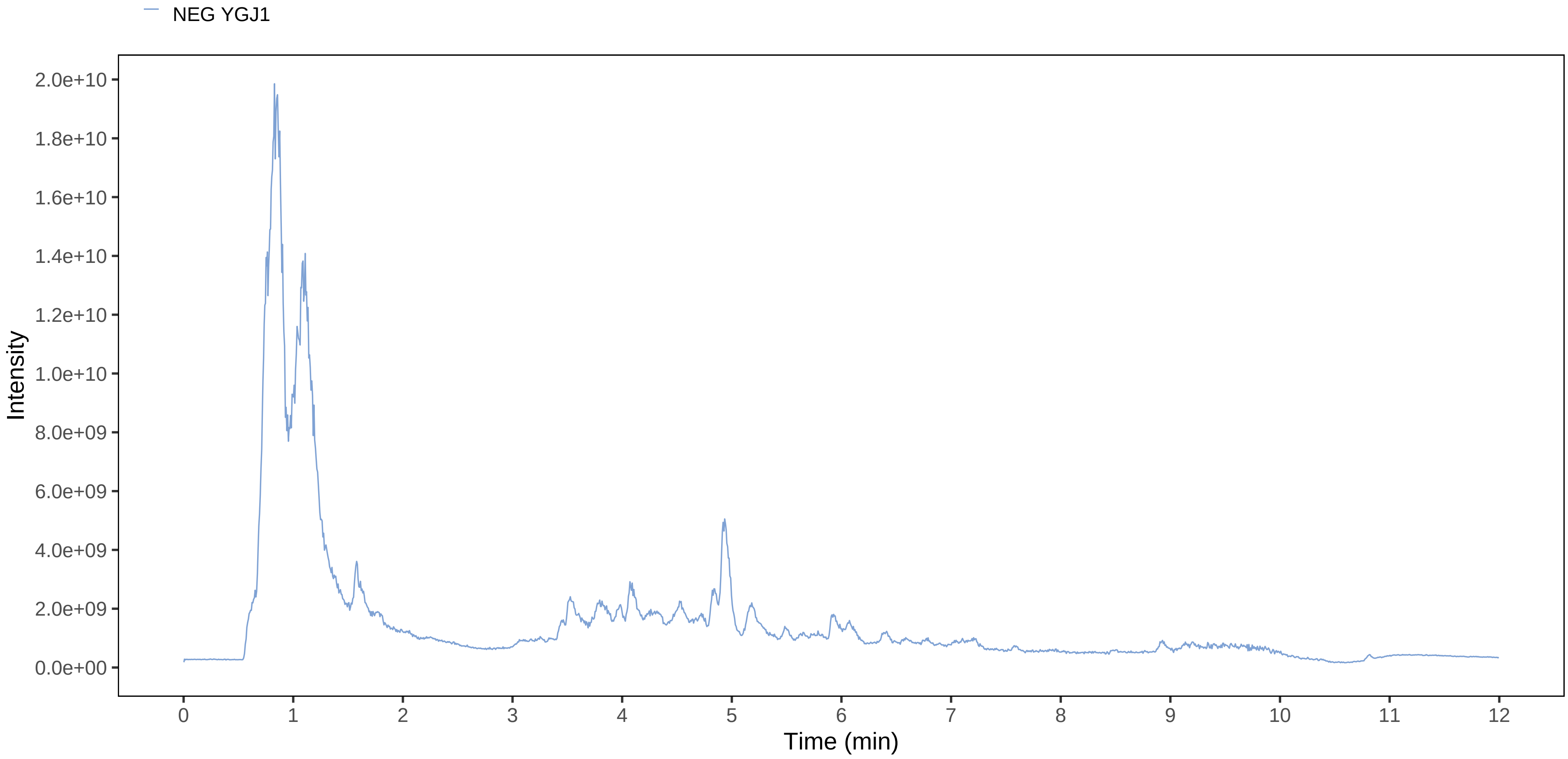
NEG YGJ1
Intensity
2.0e+10
1.8e+10
1.6e+10
1.4e+10
1.2e+10
1.0e+10
8.0e+09
6.0e+09
4.0e+09
2.0e+09
0.0e+00
0
1
2
3
4
5
6
7
8
9
10
11
12
Time (min)

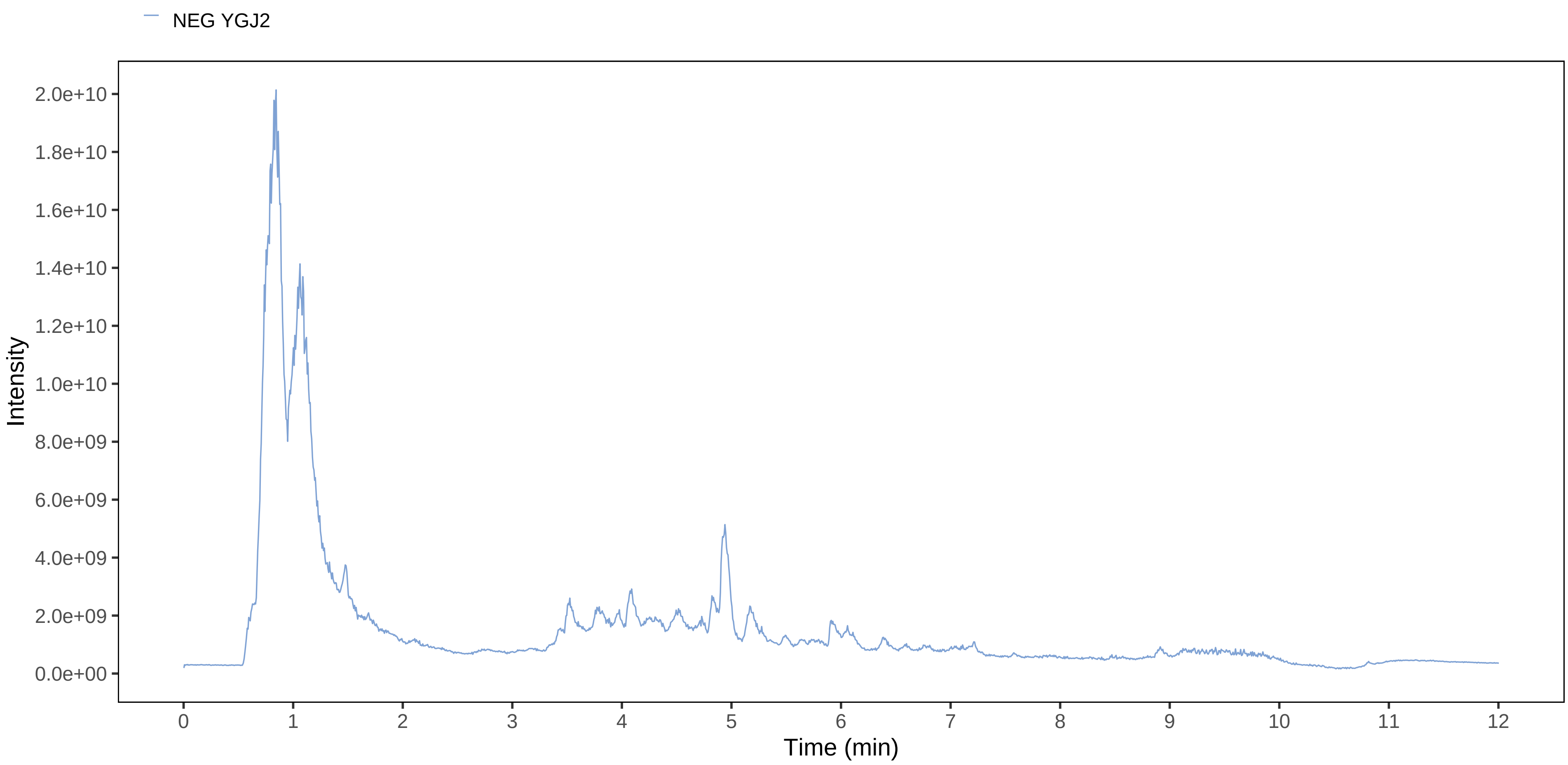
NEG YGJ2
Intensity
2.0e+10
1.8e+10
1.6e+10
1.4e+10
1.2e+10
1.0e+10
8.0e+09
6.0e+09
4.0e+09
2.0e+09
0.0e+00
0
1
2
3
4
5
6
7
8
9
10
11
12
Time (min)

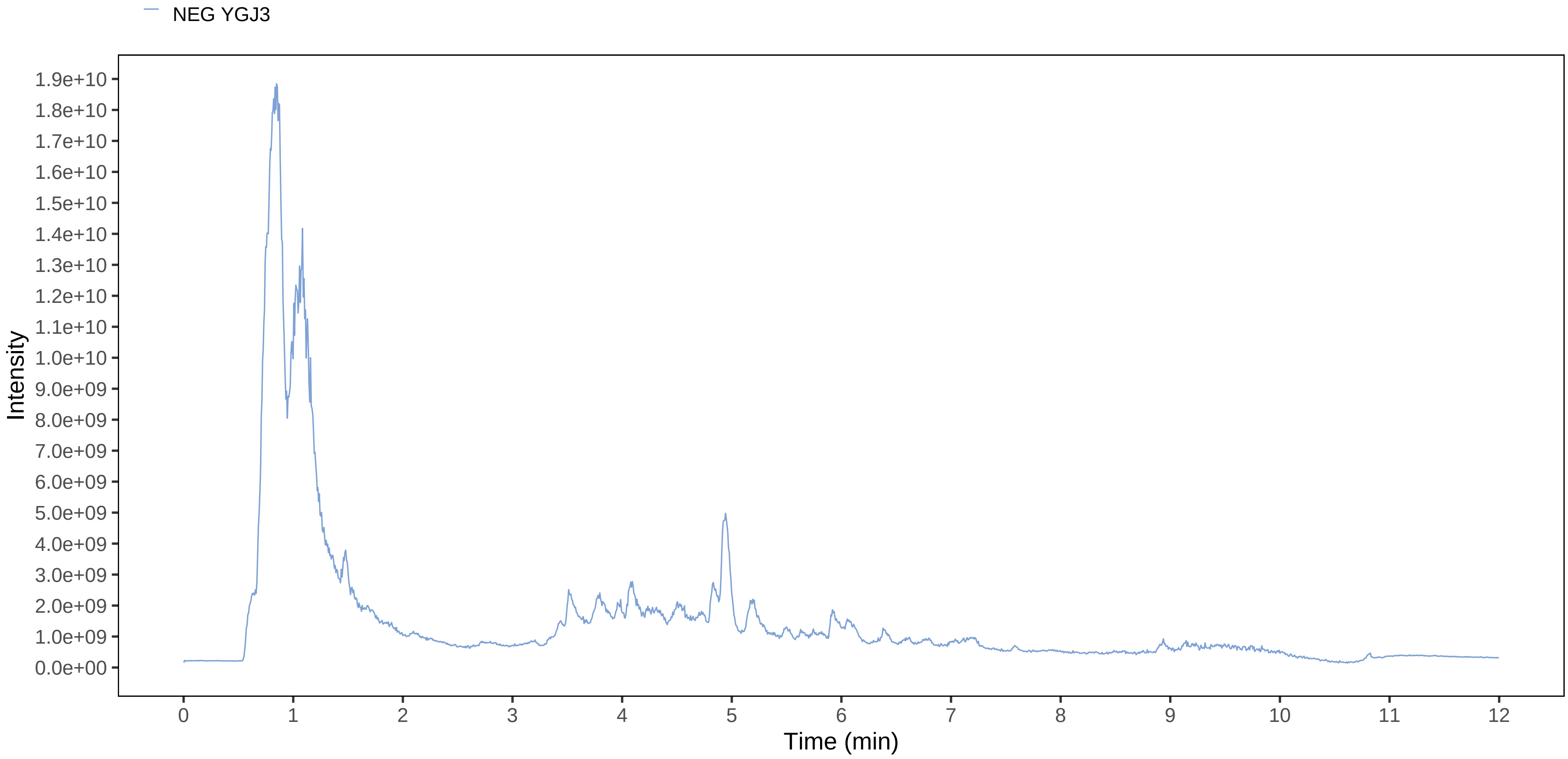

NEG YGJ3
Intensity
Time (min)
1.9e+10
1.8e+10
1.7e+10
1.6e+10
1.5e+10
1.4e+10
1.3e+10
1.2e+10
1.1e+10
1.0e+10
9.0e+09
8.0e+09
7.0e+09
6.0e+09
5.0e+09
4.0e+09
3.0e+09
2.0e+09
1.0e+09
0.0e+00
0
1
2
3
4
5
6
7
8
9
10
11
12

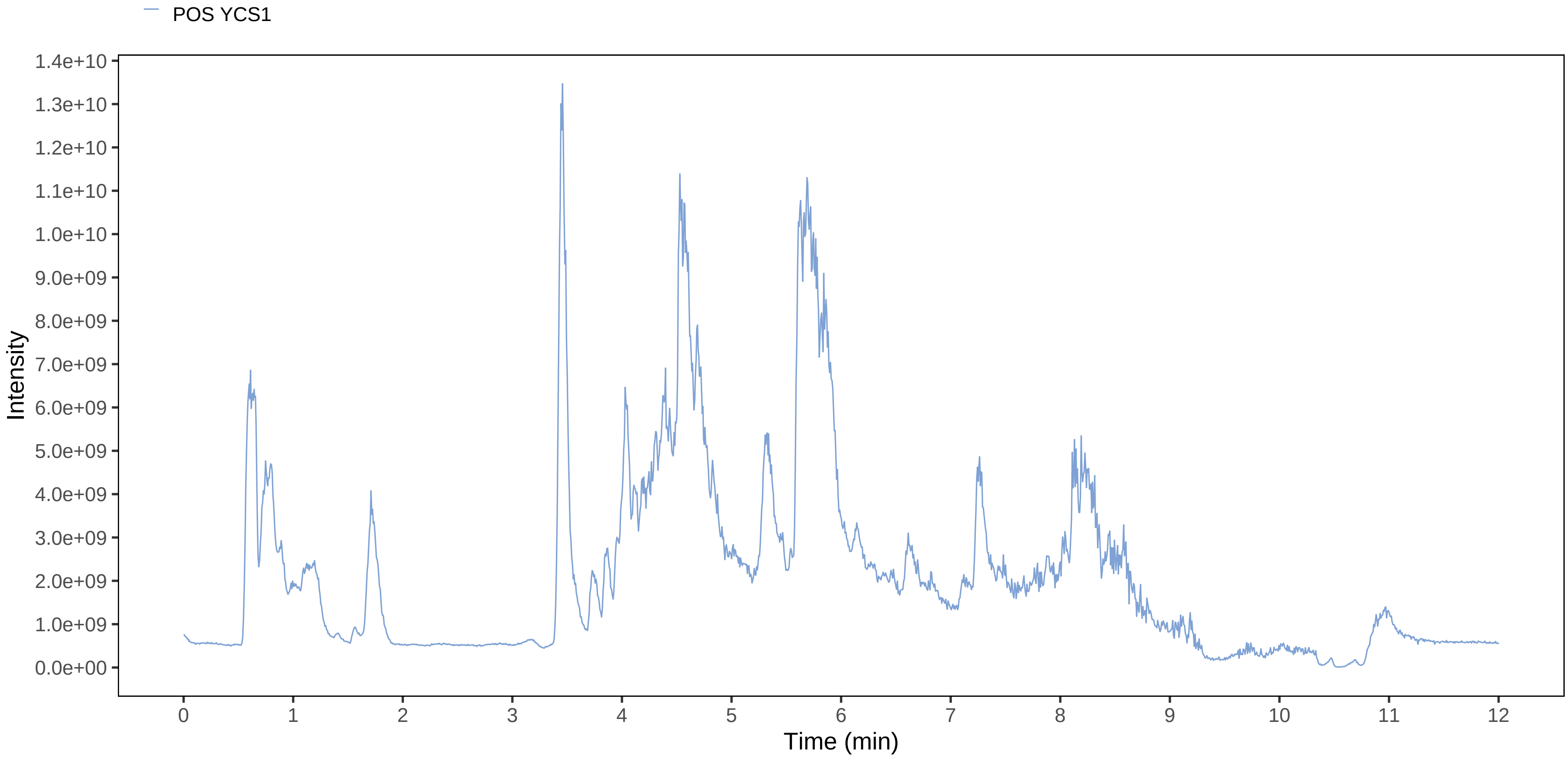

POS YCS1
Intensity
1.4e+10
1.3e+10
1.2e+10
1.1e+10
1.0e+10
9.0e+09
8.0e+09
7.0e+09
6.0e+09
5.0e+09
4.0e+09
3.0e+09
2.0e+09
1.0e+09
0.0e+00
0
1
2
3
4
5
6
7
8
9
10
11
12
Time (min)

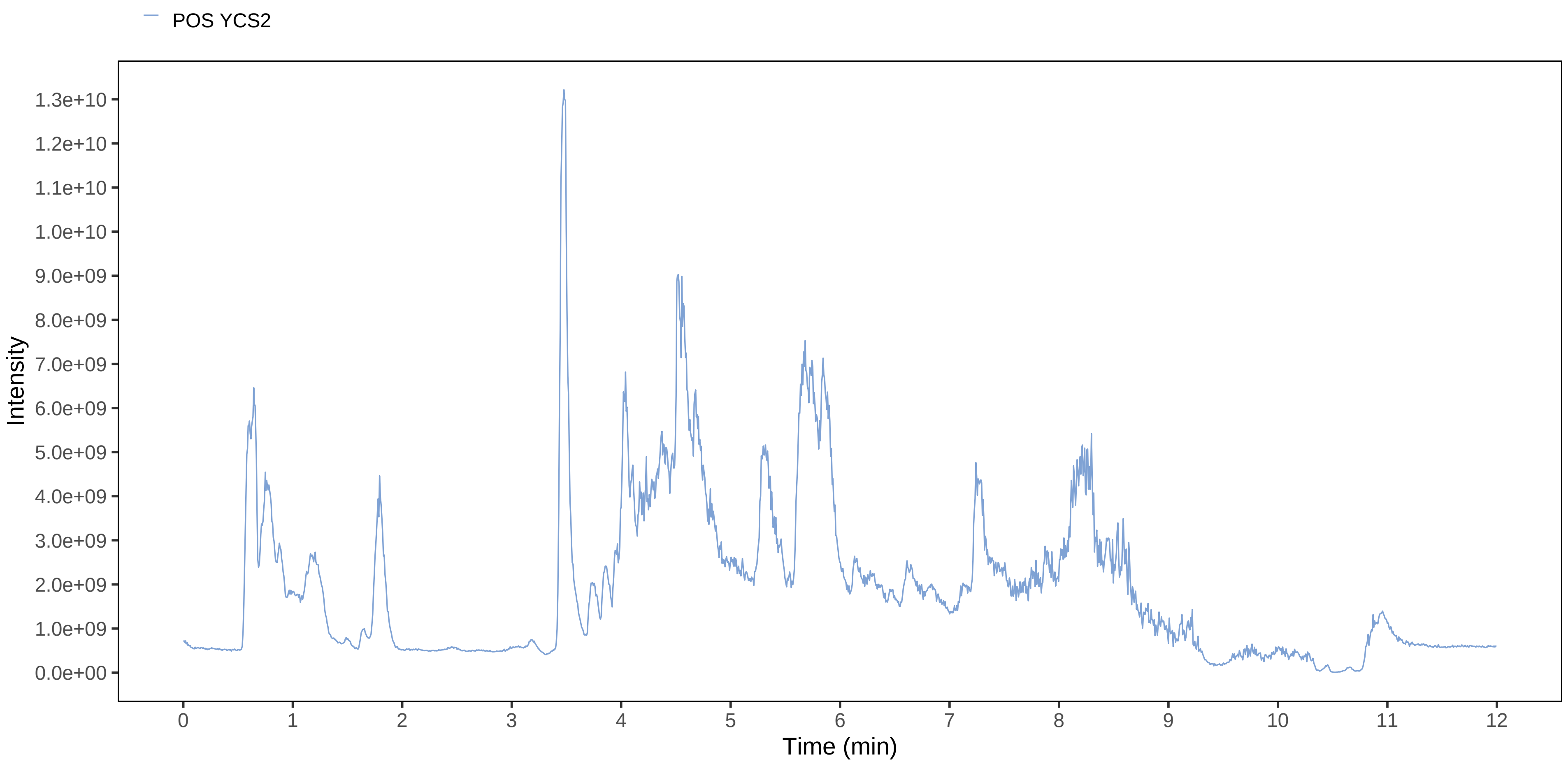

POS YCS2
Intensity
1.3e+10
1.2e+10
1.1e+10
1.0e+10
9.0e+09
8.0e+09
7.0e+09
6.0e+09
5.0e+09
4.0e+09
3.0e+09
2.0e+09
1.0e+09
0.0e+00
0
1
2
3
4
5
6
7
8
9
10
11
12
Time (min)

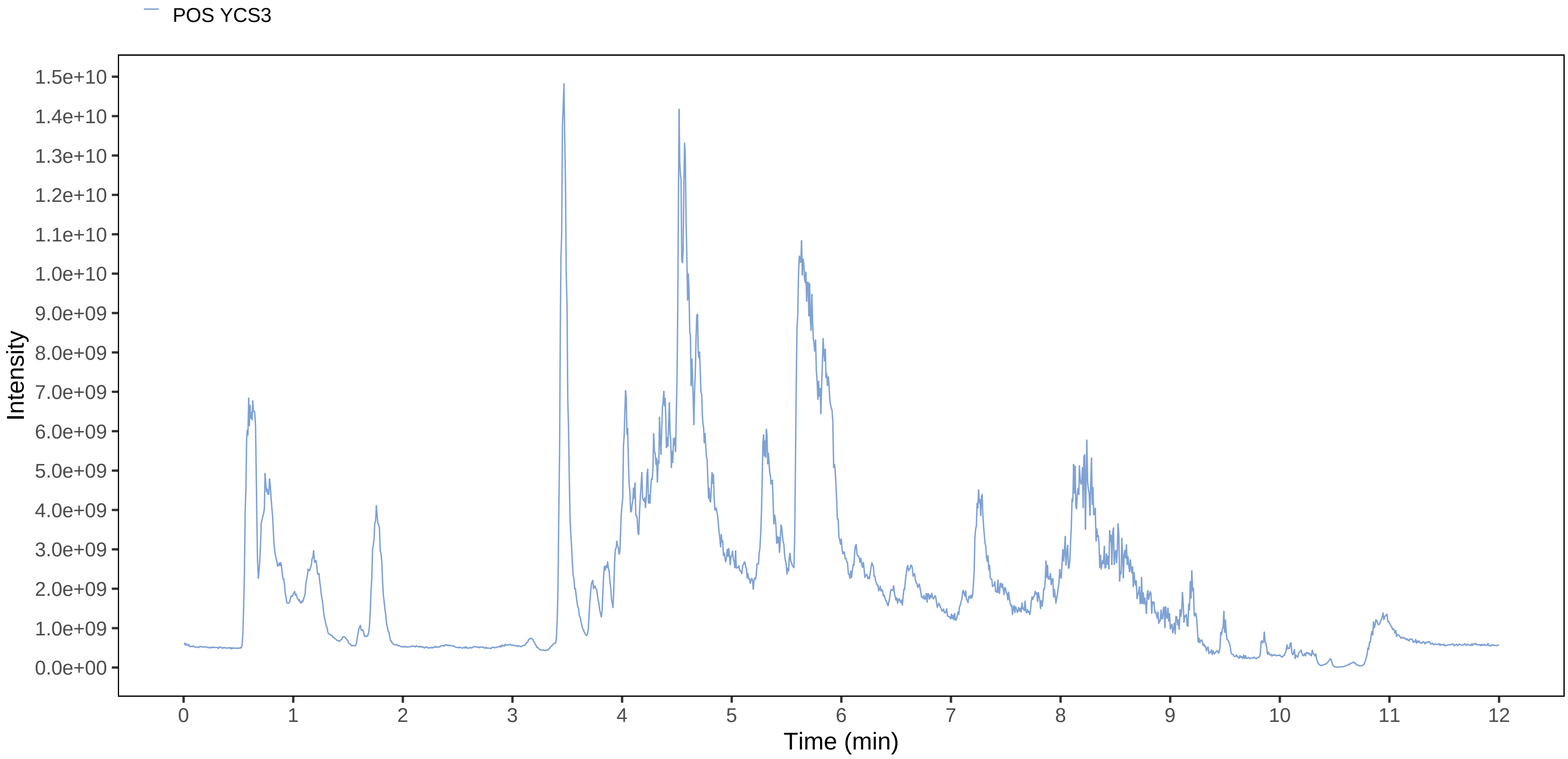
POS YCS3
Intensity
1.5e+10
1.4e+10
1.3e+10
1.2e+10
1.1e+10
1.0e+10
9.0e+09
8.0e+09
7.0e+09
6.0e+09
5.0e+09
4.0e+09
3.0e+09
2.0e+09
1.0e+09
0.0e+00
0
1
2
3
4
5
6
7
8
9
10
11
12
Time (min)

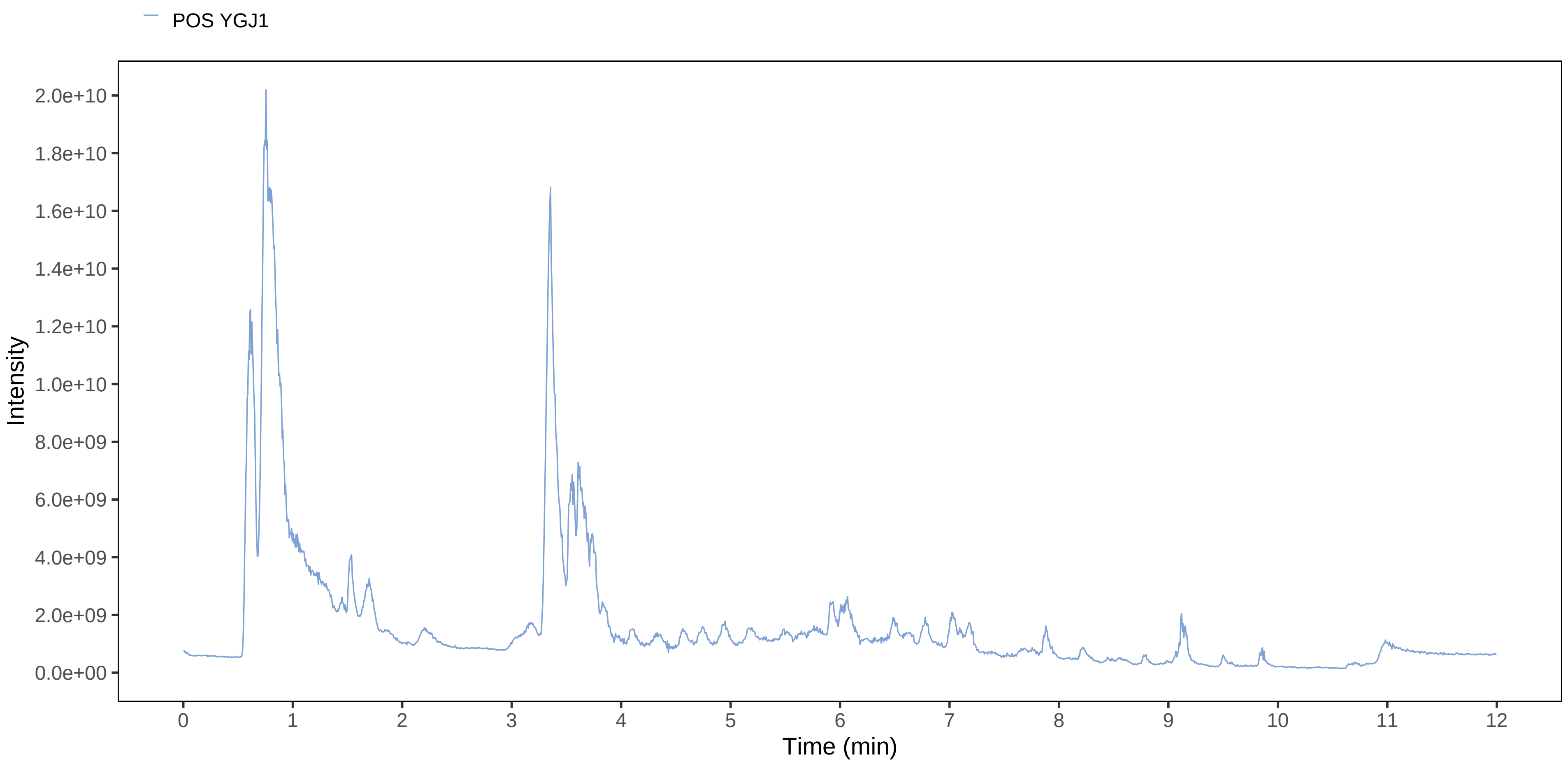
POS YGJ1
Intensity
2.0e+10
1.8e+10
1.6e+10
1.4e+10
1.2e+10
1.0e+10
8.0e+09
6.0e+09
4.0e+09
2.0e+09
0.0e+00
0
1
2
3
4
5
6
7
8
9
10
11
12
Time (min)

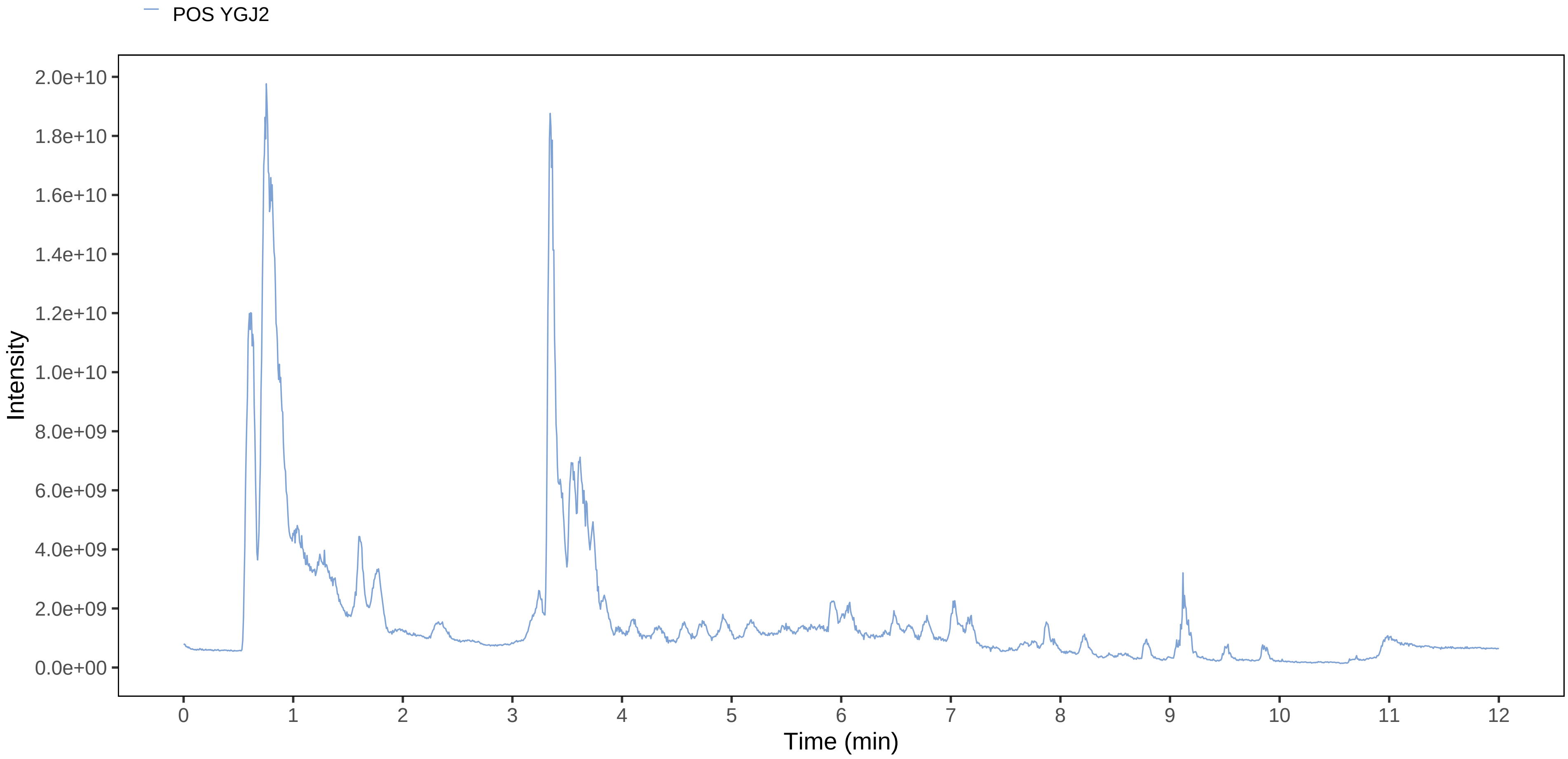

POS YGJ2
Intensity
2.0e+10
1.8e+10
1.6e+10
1.4e+10
1.2e+10
1.0e+10
8.0e+09
6.0e+09
4.0e+09
2.0e+09
0.0e+00
0
1
2
3
4
5
6
7
8
9
10
11
12
Time (min)

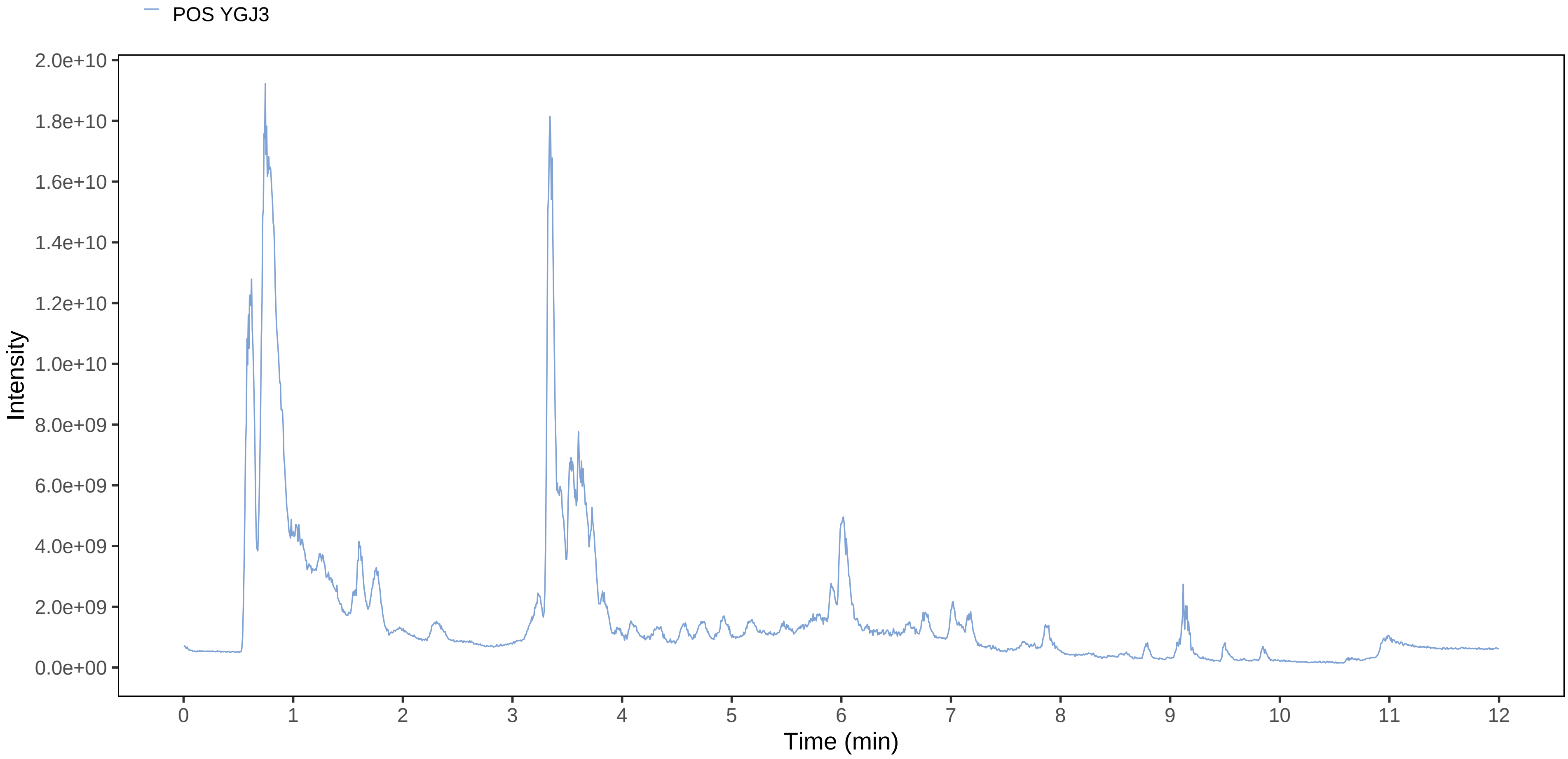
POS YGJ3
Intensity
2.0e+10
1.8e+10
1.6e+10
1.4e+10
1.2e+10
1.0e+10
8.0e+09
6.0e+09
4.0e+09
2.0e+09
0.0e+00
0
1
2
3
4
5
6
7
8
9
10
11
12
Time (min)
