# Supplementary material for: Yiguanjian alleviates ocular surface inflammation and lacrimal gland immune dysregulation in experimental dry eye disease: evidence from in vivo and in vitro models
Source: Front Med (Lausanne). 2026 Sep 28;13:1915720. doi: 10.3389/fmed.2026.1915720 (PMC13654703; doi:10.3389/fmed.2026.1915720)
Supplement: Supplementary file 2 [file Table1.docx]

**Supplementary Table S1. Primer sequences used for RT-qPCR analysis of rat lacrimal gland tissues in the in vivo experiment**

| **Gene** | **Primer** | **Sequence (5′–3′)** | **Product size (bp)** |
| --- | --- | --- | --- |
| ACTIN | Forward | CACCGCAAATGCTTCTAGGC | 186 |
|  | Reverse | TGTCACCTTCACCGTTCCAG |  |
| RORγt | Forward | CTGCAAGACTCATCGCCAAAG | 83 |
|  | Reverse | TTTCCACATGCTGGCTACACA |  |
| IL-17A | Forward | ACTACAACCGATCCACCTCAC | 83 |
|  | Reverse | ACTTTGCCTCCCAGATCACAG |  |
| Foxp3 | Forward | CTGGCAAATGGTGTCTGCAAGT | 107 |
|  | Reverse | CTGCCCTTCTCATCCAGAAGATG |  |
| IL-10 | Forward | GGTTGCCAAGCCTTATCGGAAATG | 150 |
|  | Reverse | GCCGCATCCTGAGGGTCTTC |  |
