# Supplementary material for: Yiguanjian alleviates ocular surface inflammation and lacrimal gland immune dysregulation in experimental dry eye disease: evidence from in vivo and in vitro models
Source: Front Med (Lausanne). 2026 Sep 28;13:1915720. doi: 10.3389/fmed.2026.1915720 (PMC13654703; doi:10.3389/fmed.2026.1915720)
Supplement: Supplementary file 3 [file Table2.docx]

**Supplementary Table S2. Primer sequences used for RT-qPCR analysis of CD4⁺ T cells and lacrimal gland epithelial cells in the in vitro experiment**

| **Gene** | **Primer** | **Sequence (5′–3′)** | **Product size (bp)** |
| --- | --- | --- | --- |
| GAPDH | Forward | GACAACTTTGGCATCGTGGA | 133 |
|  | Reverse | ATGCAGGGATGATGTTCTGG |  |
| RORγt | Forward | CACAGAGACACCACCGAACA | 300 |
|  | Reverse | GGCCGAACTTGACAGCATCT |  |
| IL-17A | Forward | GACTCTGAGCCGCAATGA | 108 |
|  | Reverse | ATGTGGTGGTCCAACTTCC |  |
| IFN-γ | Forward | AACTGGCAAAAGGACGGTAA | 207 |
|  | Reverse | TGGGTTGTTCACCTCGAACT |  |
| Foxp3 | Forward | ATGTTCGCCTACTTCAGAA | 122 |
|  | Reverse | TCATCTACGGTCCACACT |  |
| TGF-β | Forward | GCTGAACCAAGGAGACGGAA | 343 |
|  | Reverse | GAAGTTGGCATGGTAGCCCT |  |
| IL-10 | Forward | GGCTCAGCACTGCTATGTTGCC | 116 |
|  | Reverse | AGCATGTGGGTCTGGCTGACTG |  |
